# Tooth Size Variation in Pinniped Dentitions

**DOI:** 10.1371/journal.pone.0137100

**Published:** 2015-08-28

**Authors:** Mieczyslaw Wolsan, Satoshi Suzuki, Masakazu Asahara, Masaharu Motokawa

**Affiliations:** 1 The Kyoto University Museum, Kyoto University, Kyoto, Japan; 2 Museum and Institute of Zoology, Polish Academy of Sciences, Warsaw, Poland; 3 Department of Zoology, Graduate School of Science, Kyoto University, Kyoto, Japan; NYIT College of Osteopathic Medicine, UNITED STATES

## Abstract

It is contentious whether size variation among mammalian teeth is heterogeneous or homogeneous, whether the coefficient of variation is reliable, and whether the standard deviation of log-transformed data and the residual of standard deviation on mean variable size are useful replacements for the coefficient of variation. Most studies of tooth size variation have been on mammals with complex-crowned teeth, with relatively little attention paid to taxa with simple-crowned teeth, such as Pinnipedia. To fill this gap in knowledge and to resolve the existing controversies, we explored the variation of linear size variables (length and width) for all teeth from complete permanent dentitions of four pinniped species, two phocids (*Histriophoca fasciata*, *Phoca largha*) and two otariids (*Callorhinus ursinus*, *Eumetopias jubatus*). Size variation among these teeth was mostly heterogeneous both along the toothrow and among species. The incisors, canines, and mesial and distal postcanines were often relatively highly variable. The levels of overall dental size variation ranged from relatively low as in land carnivorans (*Phoca largha* and both otariids) to high (*Histriophoca fasciata*). Sexual size dimorphism varied among teeth and among species, with teeth being, on average, larger in males than in females. This dimorphism was more pronounced, and the canines were larger and more dimorphic relative to other teeth in the otariids than in the phocids. The coefficient of variation quantified variation reliably in most cases. The standard deviation of log-transformed data was redundant with the coefficient of variation. The residual of standard deviation on mean variable size was inaccurate when size variation was considerably heterogeneous among the compared variables, and was incomparable between species and between sexes. The existing hypotheses invoking developmental fields, occlusal complexity, and the relative timing of tooth formation and sexually dimorphic hormonal activity do not adequately explain the differential size variation along the pinniped toothrow.

## Introduction

Variation is a prerequisite for evolution by natural selection. Therefore, variation has been the focus of considerable biological research for over 150 years [1–3]. No characterization of a taxon, population, organism, or organ can be complete without characterizing its variation.

Dentition is of fundamental importance for the study of mammalian evolution. This is because teeth are highly informative of a mammal’s taxonomic identity, ecological adaptation, and phylogenetic relationships, and because they are chiefly inorganic, which makes them durable and relatively abundant in the fossil record [4, 5]. The size of a tooth crown is fixed by the cessation of enamel apposition before tooth eruption, offering a correlate to body size [6–9], which in turn correlates with many aspects of an animal’s life history and ecology [10, 11].

A common index of variation is CV, which is the ratio of SD to mean (these and other statistical abbreviations used in the paper are explained in Table 1). Because ME tends to uniformly contribute to SD, CV is usually negatively correlated with mean variable size and may become artificially high if the ME of a variable is high and the variable is small [12]. To counteract this bias in CV, two alternative measures of variation (SDL and RSD) were proposed [12]. SDL and RSD have been used in several studies [12–15], but the usefulness of these indices and the reliability of CV, though discussed [12–17], have remained largely unexplored.

**Table 1:** Explanation of Statistical Abbreviations and Symbols.

Abbreviation or symbol	Name and/or definition
CV	Coefficient of variation, or the ratio of the standard deviation of a variable to the arithmetic mean of that variable, multiplied by 100
M/F	Sexual size dimorphism index, or the ratio of the arithmetic mean of a variable in males to the arithmetic mean of that variable in females
ME	Measurement error, or [*s* ^2^_ind_/(*s* ^2^_ind_ + *s* ^2^_pop_)] × 100, where *s* ^2^_ind_ is the variance of the repeated measurements of a variable in a single individual, and *s* ^2^_pop_ is the variance of the measurements of that variable among all individuals in the sample [12]
mean	Arithmetic mean
*n*	Sample size
*P*	Probability of wrongly rejecting the null hypothesis of no difference or relationship
*r*	Pearson’s product moment correlation coefficient
*r*_*s*_	Spearman’s rank correlation coefficient
RSD	Residual of the standard deviation of a variable regressed on the arithmetic mean of that variable
SD	Standard deviation
SDL	Standard deviation of log-transformed data

An array of studies have documented (using CV) patterns of variation in tooth size within mammalian dentitions, with certain teeth being consistently more or less variable than others (e.g., [13, 14, 18–28]). These patterns have been explained in terms of the relative position of teeth in a developmental [29] (morphogenetic [30] or growth [31]) field [18, 19, 24, 32], the relative occlusal complexity of tooth crowns [13, 15, 20, 21], or the relative timing of tooth formation and sexually dimorphic hormonal activity [12–15, 18]. The hypothesis invoking relative tooth position in a developmental field assumes that the level of variation in the size of a tooth depends on the position of that tooth in an incisor, canine, or postcanine field and that teeth in the center of such a developmental field are less variable than those at the periphery of that field [18, 19]. The hypothesis invoking relative tooth occlusal complexity postulates that the level of size variation in teeth is inversely proportional to their occlusal complexity [20]. Finally, the hypothesis invoking the relative timing of tooth formation and sexually dimorphic hormonal activity proposes that teeth forming before the onset of sex-linked differentiation in hormonal balance are less variable than those forming during sexually differentiated growth and that the latter teeth show progressively higher variation indices when males and females are pooled [18].

Polly [12] argued that the observed patterns of variation were the effect of size-related bias in CV and that size variation among mammalian teeth is indeed relatively homogeneous both within and among species. The generality of this conclusion was undermined by subsequent studies [14–17], which showed that the patterns of tooth size variation seen in the dentitions of various land carnivorans were not entirely generated by bias in CV.

**Fig 1:**
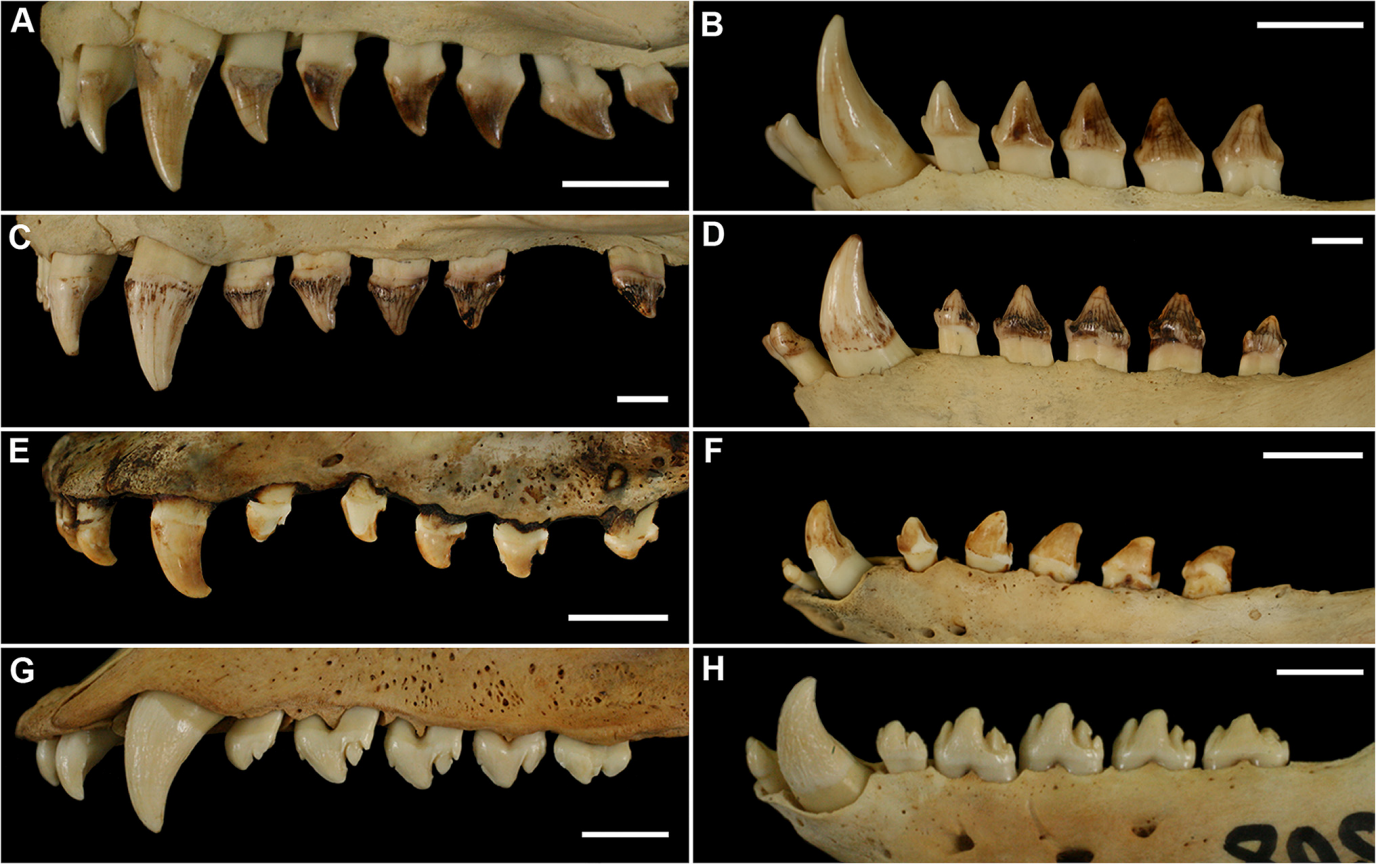
Vestibular Profiles of Upper (Left) and Lower (Right) Pinniped Permanent Dentitions. A, B, northern fur seal (*Callorhinus ursinus*), female, KUZ M10142 (KUZ, Kyoto University Museum); C, D, Steller sea lion (*Eumetopias jubatus*), female, KUZ M9290; E, F, ribbon seal (*Histriophoca fasciata*), male, KUZ M9575; G, H, spotted seal (*Phoca largha*), female, KUZ M9465, reversed mirror image. Scale bars equal 1 cm.

Variation in tooth size has been studied in various mammalian clades, mainly in primates (e.g., [19, 20, 22, 23, 26–28, 33]) and canids (e.g., [12–14, 16, 21, 24, 34–39]). Most studies have been on taxa with complex-crowned teeth, which prevail among mammals, with relatively little attention paid to taxa with simple-crowned teeth, such as Pinnipedia, a clade of aquatic (mostly marine) carnivorans (Fig 1). The only study of tooth size variation using a large sample from a pinniped species to date has been that of Miller et al. [40], who investigated the ringed seal’s (*Pusa hispida*) third lower premolar and the harp seal’s (*Pagophilus groenlandicus*) lower postcanines. These authors found that CVs for size variables of these teeth were higher than those in the compared land carnivorans and therefore hypothesized that the postcanines of pinnipeds are more variable in size than those of land carnivorans due to evolutionary simplification of morphology via selective release.

The purpose of this study was fourfold: (1) to evaluate the reliability and usefulness of CV, SDL, and RSD; (2) to describe and compare the variation of tooth size within and among species of Pinnipedia representing its two major extant clades, Otariidae and Phocidae; (3) to compare the variation of tooth size between pinnipeds and land carnivorans; and (4) to test hypotheses related to tooth size variation. Specifically, we test the following hypotheses: (1) that CV is not a reliable measure of variation and that SDL and RSD are more reliable [12]; (2) that size variation among mammalian teeth is relatively homogeneous both within and among species except highly variable canines in some species [12]; (3) that the postcanines of pinnipeds are more variable in size than those of land carnivorans due to evolutionary simplification of morphology [40]; (4) that the level of variation in the size of a tooth depends on the position of that tooth in an incisor, canine, or postcanine developmental field, with teeth in the center of that field being less variable than those at the periphery [18, 19]; (5) that the level of size variation in teeth is inversely proportional to their occlusal complexity [20]; and (6) that teeth forming before the onset of sex-linked differentiation in hormonal balance are less variable than those forming during sexually differentiated growth, with the latter teeth showing progressively higher variation indices when males and females are pooled [18].

## Materials and Methods

Measurements were collected from permanent dentitions in skeletonized specimens of four species representing two families of Pinnipedia from the collections of five institutions (Fig 1, Tables 2 and 3). According to the collection records, these specimens derived from wild animals on and around the Japanese Islands. For each specimen, two linear size variables (length and width; Table 4) were measured on all teeth of one body side, left or right, depending on the state of preservation. All measurements were taken with digital calipers to the nearest 0.01 mm. ME was assessed by measuring variables 10 times for one randomly selected specimen. Size variation was quantified using three indices: CV, SDL, and RSD. Sexual size dimorphism was evaluated with M/F [41]. Data on the complete sequence of tooth eruption were taken from the relevant literature (these data were available only for *Callorhinus ursinus*). Statistical analyses were performed in R versions 2.13.2 and 3.0.2 [42].

**Table 2:** Measured Specimens.

Family	Species	Males	Females
Otariidae	*Callorhinus ursinus* (Linnaeus, 1758) [43]	HUNHM 9893, 11560, 13349; KUZ M10017, M10019, M10023, M10027, M10031, M10033, M10034, M10043, M10051, M10067–M10069, M10092–M10095, M10098, M10099, M10101–M10103, M10106–M10110, M10113, M10116, M10120, M10121, M10124, M10126, M10141, M10279, M10281, M10372; NSMT KK70, KK162, M1994, M1996	KUZ M10018, M10020, M10021, M10024, M10029, M10035, M10038, M10040, M10041, M10044–M10046, M10048–M10050, M10053, M10055, M10056, M10058–M10062, M10064, M10065, M10081, M10082, M10085, M10087, M10089, M10090, M10114, M10115, M10119, M10123, M10125, M10127–M10129, M10131, M10132, M10135, M10137, M10138, M10140, M10142, M10144, M10147–M10149, M10151, M10154, M10155, M10157; NSMT KK8, KK10, KK22, KK151, M1995
	*Eumetopias jubatus* (Schreber, 1776) [44]	HUM 1, 3, 4, 6, 13, 16, 21, 22, 31; KUZ M9286, M9436, M9438, M9581, M9586, M9588, M9590, M9594, M9985, M9987–M9991, M9993, M9994, M10001, M10004–M10006; NSMT KK42, KK122	HUNHM 13313, 13351; KUZ M9290, M9291, M9295, M9297, M9309, M9311, M9437, M9440–M9442, M9968; NSMT KK55, KK62, KK67, KK125, KK131, KK135, KK139, KK146, KK154, KK156, KK158, M17123, M24719, M24723, M24724, N113, PO136
Phocidae	*Histriophoca fasciata* (Zimmermann, 1783) [45]	HUNHM 17216, 17217, 17246, 17249; KUZ M9397, M9398, M9401, M9407, M9409, M9425, M9454, M9460, M9466, M9493, M9559, M9572, M9575, M9577, M9605, M9608, M9619, M9639, M9640, M9645, M9647, M9657, M9659, M9665, M9671, M9680, M9682, M9685, M9688, M9711, M9720, M9724, M9770, M9771, M9801, M9808, M9813, M9823, M9856, M9858, M9861, M9875, M9876, M9884, M9976, M10370; TUA 349, NK502, P265, P274, P275, P291, P297, P300, RK501, RK502, RK505, RK507	HUM 10, 44; HUNHM 17222, 47748, 47749; KUZ M9312, M9314, M9325, M9334, M9338, M9341, M9342, M9411, M9427, M9453, M9469, M9473, M9482, M9483, M9500, M9501, M9550, M9556, M9569, M9571, M9574, M9607, M9617, M9621, M9646, M9653, M9654, M9660, M9663, M9667, M9668, M9670, M9674, M9677–M9679, M9684, M9687, M9691, M9692, M9708, M9712–M9715, M9718, M9755, M9756, M9763, M9766, M9768, M9769, M9772, M9809, M9811, M9826–M9829, M9831, M9832, M9835, M9836, M9857, M9859, M9863, M9870–M9872, M9877–M9879; TUA 348b, P269, P279, P294, P295, P301, RK509, RK511, RK512
	*Phoca largha* Pallas, 1811 [46]	HUNHM 13325, 13326; KUZ M9277, M9279, M9336, M9413, M9415, M9457, M9533, M9548, M9610, M9623, M9741, M9745, M9851, M9902, M10342; NSMT M24771, M29787; TUA AbG701, BG902, BG905, EG5101, G18, G21, G29, HAG604, HAG607, HAG608, HAG612, NG301, NG304, NG305, NG505, NG506, NG508–NG510, NoG911, ReG909, ReG911, ReG1004, ReG1007, ReG1008, ReG1010, RG502–RG506, RG508–RG510, RG512, RG513, RG516, RG517, RG520, RG522, RG526, RG530, RG708, RG710, RG804, RG805, RG913, RG918–RG920, RG925, YG401–YG404, YG502, YG503, YG601, YG701, YG704, YG906	KUZ M9264, M9348, M9465, M9499, M9502, M9505, M9507, M9537, M9749, M9784, M9868; NSMT M28385; TUA AbG702, AbG703, AbG902, AG2, BG903, BG904, BG906, G2, G4, G23, HAG601, HAG602, HAG605, HAG606, HAG609, HAG611, NG302, NG303, NG306, NG504, ReG908, ReG912, ReG1001, ReG1002, ReG1006, ReG1009, ReG1011, RG523–RG525, RG528, RG529, RG533, RG534, RG712, RG801, RG802, RG901, RG903, RG922, RK510, YG405, YG501, YG507, YG602, YG603, YG702, YG703

Institutional abbreviations are explained in Table 3.

**Table 3:** Institutional Abbreviations.

Abbreviation	Name
HUM	Hokkaido University Museum, Hokkaido University, Sapporo, Japan
HUNHM	Botanic Garden, Hokkaido University, Sapporo, Japan
KUZ	Kyoto University Museum, Kyoto University, Kyoto, Japan
NSMT	National Museum of Nature and Science, Tokyo, Japan
TUA	Laboratory of Aquatic Management, Department of Aqua Bioscience and Industry, Faculty of Bioindustry, Tokyo University of Agriculture, Abashiri, Japan

**Table 4:** Tooth Size Variables.

Abbreviation	Name	Definition
LC^1^	Length of C^1^	Longest linear mesiodistal distance on the crown of C^1^
LC_1_	Length of C_1_	Longest linear mesiodistal distance on the crown of C_1_
LI^1^	Length of I^1^	Longest linear mesiodistal distance on the crown of I^1^
LI^2^	Length of I^2^	Longest linear mesiodistal distance on the crown of I^2^
LI_2_	Length of I_2_	Longest linear mesiodistal distance on the crown of I_2_
LI^3^	Length of I^3^	Longest linear mesiodistal distance on the crown of I^3^
LI_3_	Length of I_3_	Longest linear mesiodistal distance on the crown of I_3_
LM^1^	Length of M^1^	Longest linear mesiodistal distance on the crown of M^1^
LM_1_	Length of M_1_	Longest linear mesiodistal distance on the crown of M_1_
LM^2^	Length of M^2^	Longest linear mesiodistal distance on the crown of M^2^
LP^1^	Length of P^1^	Longest linear mesiodistal distance on the crown of P^1^
LP_1_	Length of P_1_	Longest linear mesiodistal distance on the crown of P_1_
LP^2^	Length of P^2^	Longest linear mesiodistal distance on the crown of P^2^
LP_2_	Length of P_2_	Longest linear mesiodistal distance on the crown of P_2_
LP^3^	Length of P^3^	Longest linear mesiodistal distance on the crown of P^3^
LP_3_	Length of P_3_	Longest linear mesiodistal distance on the crown of P_3_
LP^4^	Length of P^4^	Longest linear mesiodistal distance on the crown of P^4^
LP_4_	Length of P_4_	Longest linear mesiodistal distance on the crown of P_4_
WC^1^	Width of C^1^	Longest linear vestibulolingual distance on the crown of C^1^ perpendicular to LC^1^
WC_1_	Width of C_1_	Longest linear vestibulolingual distance on the crown of C_1_ perpendicular to LC_1_
WI^1^	Width of I^1^	Longest linear vestibulolingual distance on the crown of I^1^ perpendicular to LI^1^
WI^2^	Width of I^2^	Longest linear vestibulolingual distance on the crown of I^2^ perpendicular to LI^2^
WI_2_	Width of I_2_	Longest linear vestibulolingual distance on the crown of I_2_ perpendicular to LI_2_
WI^3^	Width of I^3^	Longest linear vestibulolingual distance on the crown of I^3^ perpendicular to LI^3^
WI_3_	Width of I_3_	Longest linear vestibulolingual distance on the crown of I_3_ perpendicular to LI_3_
WM^1^	Width of M^1^	Longest linear vestibulolingual distance on the crown of M^1^ perpendicular to LM^1^
WM_1_	Width of M_1_	Longest linear vestibulolingual distance on the crown of M_1_ perpendicular to LM_1_
WM^2^	Width of M^2^	Longest linear vestibulolingual distance on the crown of M^2^ perpendicular to LM^2^
WP^1^	Width of P^1^	Longest linear vestibulolingual distance on the crown of P^1^ perpendicular to LP^1^
WP_1_	Width of P_1_	Longest linear vestibulolingual distance on the crown of P_1_ perpendicular to LP_1_
WP^2^	Width of P^2^	Longest linear vestibulolingual distance on the crown of P^2^ perpendicular to LP^2^
WP_2_	Width of P_2_	Longest linear vestibulolingual distance on the crown of P_2_ perpendicular to LP_2_
WP^3^	Width of P^3^	Longest linear vestibulolingual distance on the crown of P^3^ perpendicular to LP^3^
WP_3_	Width of P_3_	Longest linear vestibulolingual distance on the crown of P_3_ perpendicular to LP_3_
WP^4^	Width of P^4^	Longest linear vestibulolingual distance on the crown of P^4^ perpendicular to LP^4^
WP_4_	Width of P_4_	Longest linear vestibulolingual distance on the crown of P_4_ perpendicular to LP_4_

Tooth symbols are explained in Table 5.

**Table 5:** Explanation of Tooth Symbols.

Symbol	Name
C^1^	Upper canine
C_1_	Lower canine
I^1^	First upper incisor
I_1_	First lower incisor
I^2^	Second upper incisor
I_2_	Second lower incisor
I^3^	Third upper incisor
I_3_	Third lower incisor
M^1^	First upper molar
M_1_	First lower molar
M^2^	Second upper molar
M_2_	Second lower molar
M_3_	Third lower molar
P^1^	First upper premolar
P_1_	First lower premolar
P^2^	Second upper premolar
P_2_	Second lower premolar
P^3^	Third upper premolar
P_3_	Third lower premolar
P^4^	Fourth upper premolar
P_4_	Fourth lower premolar

## Results

### Tooth size

**Fig 2:**
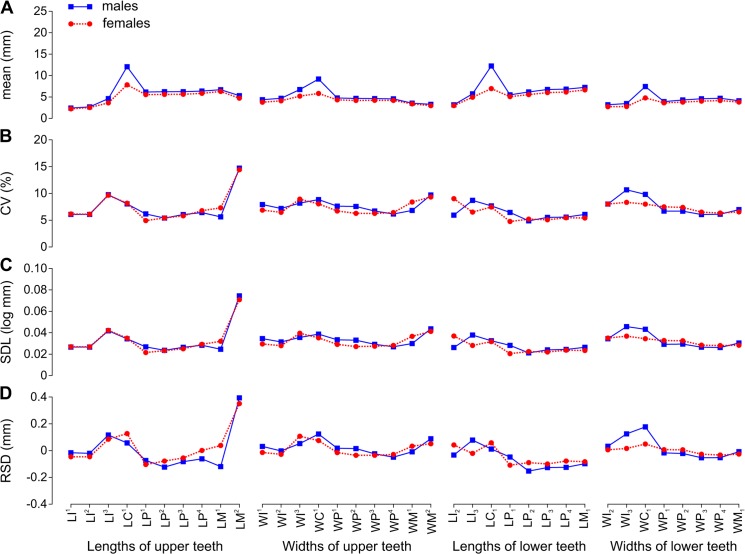
Arithmetic Mean and Variation Indices for Tooth Lengths and Widths Varied along the Toothrow and between Sexes in *Callorhinus ursinus*. A, arithmetic mean (mean); B, coefficient of variation (CV); C, standard deviation of log-transformed data (SDL); D, residual of standard deviation on arithmetic mean (RSD). Data from Table 6. Abbreviations for tooth lengths and widths are explained in Table 4.

**Fig 3:**
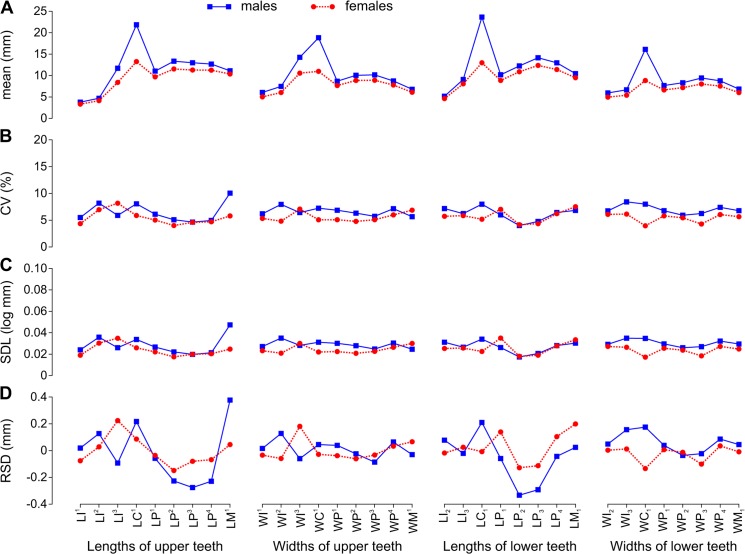
Arithmetic Mean and Variation Indices for Tooth Lengths and Widths Varied along the Toothrow and between Sexes in *Eumetopias jubatus*. A, arithmetic mean (mean); B, coefficient of variation (CV); C, standard deviation of log-transformed data (SDL); D, residual of standard deviation on arithmetic mean (RSD). Data from Table 7. Abbreviations for tooth lengths and widths are explained in Table 4.

**Fig 4:**
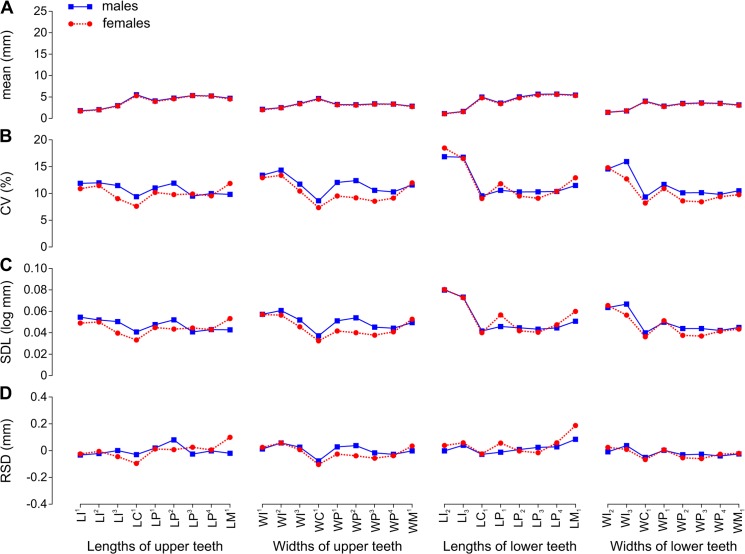
Arithmetic Mean and Variation Indices for Tooth Lengths and Widths Varied along the Toothrow and between Sexes in *Histriophoca fasciata*. A, arithmetic mean (mean); B, coefficient of variation (CV); C, standard deviation of log-transformed data (SDL); D, residual of standard deviation on arithmetic mean (RSD). Data from Table 8. Abbreviations for tooth lengths and widths are explained in Table 4.

**Fig 5:**
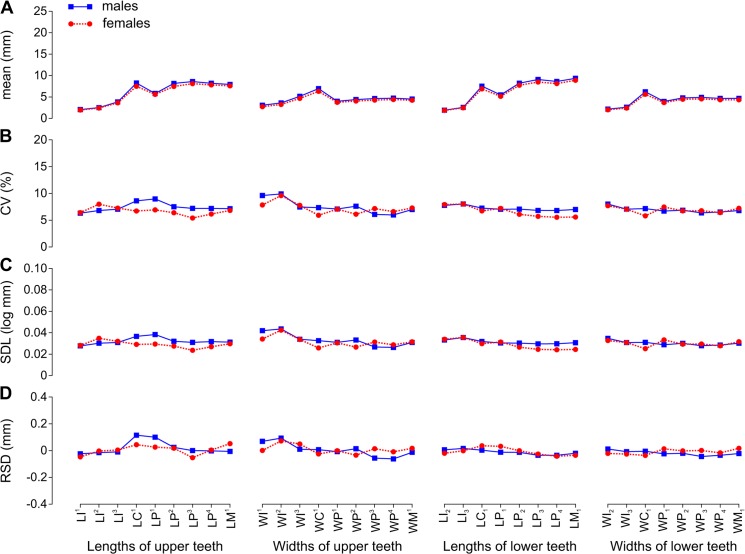
Arithmetic Mean and Variation Indices for Tooth Lengths and Widths Varied along the Toothrow and between Sexes in *Phoca largha*. A, arithmetic mean (mean); B, coefficient of variation (CV); C, standard deviation of log-transformed data (SDL); D, residual of standard deviation on arithmetic mean (RSD). Data from Table 9. Abbreviations for tooth lengths and widths are explained in Table 4.

**Fig 6:**
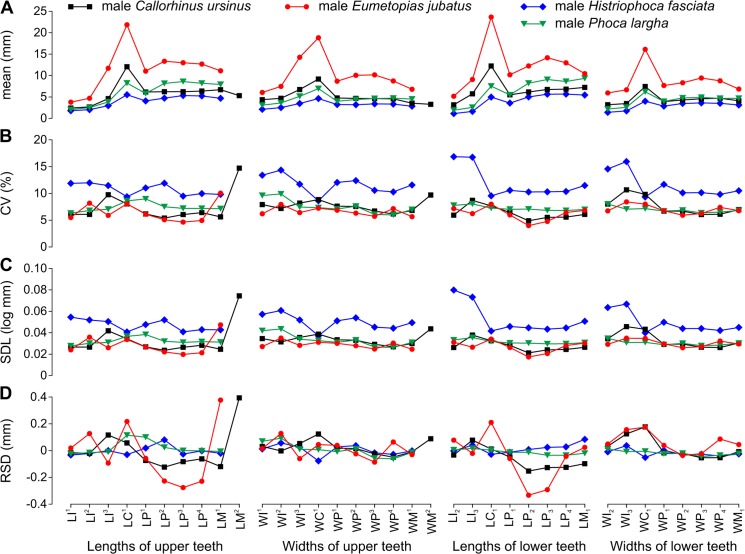
Arithmetic Mean and Variation Indices for Tooth Lengths and Widths Varied along the Toothrow and among Male Pinniped Species. A, arithmetic mean (mean); B, coefficient of variation (CV); C, standard deviation of log-transformed data (SDL); D, residual of standard deviation on arithmetic mean (RSD). Data from Tables 6–9. Abbreviations for tooth lengths and widths are explained in Table 4.

**Fig 7:**
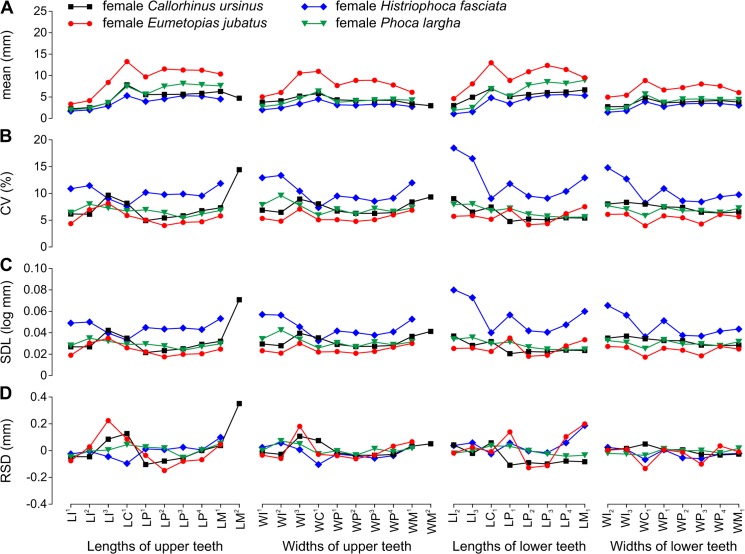
Arithmetic Mean and Variation Indices for Tooth Lengths and Widths Varied along the Toothrow and among Female Pinniped Species. A, arithmetic mean (mean); B, coefficient of variation (CV); C, standard deviation of log-transformed data (SDL); D, residual of standard deviation on arithmetic mean (RSD). Data from Tables 6–9. Abbreviations for tooth lengths and widths are explained in Table 4.

Mean variable size was 2.23–12.23 mm in *Callorhinus ursinus* (Fig 2A, Table 6), 3.33–23.64 mm in *Eumetopias jubatus* (Fig 3A, Table 7), 1.07–5.67 mm in *Histriophoca fasciata* (Fig 4A, Table 8), and 1.87–9.35 mm in *Phoca largha* (Fig 5A, Table 9). All teeth were, on average, largest in *E*. *jubatus*, smallest in *H*. *fasciata*, and intermediate in size in *C*. *ursinus* and *P*. *largha* (Figs 6A and 7A).

**Table 6:** Statistics for Tooth Lengths and Widths in *Callorhinus ursinus*.

Variable	Males, *n* = 43							Females, *n* = 59							M/F
	Range	mean	SD	ME	CV	SDL	RSD	Range	mean	SD	ME	CV	SDL	RSD	
Lengths of upper teeth															
LI^1^	2.09–2.71	2.42**	0.15	0.33	6.1	0.027	-0.016	1.88–2.52	2.23	0.14	0.24	6.2	0.027	-0.046	1.08
LI^2^	2.33–3.06	2.69**	0.16	0.28	6.1	0.027	-0.020	2.18–2.80	2.53	0.15	1.83	6.1	0.027	-0.046	1.07
LI^3^	3.86–5.62	4.61**	0.45	3.75	9.8	0.042	0.116	2.86–4.64	3.62	0.35	1.03	9.7	0.042	0.085	1.27
LC^1^	10.15–15.51	12.06**	0.97	0.13	8.0	0.034	0.057	6.87–9.29	7.84	0.64	0.24	8.2	0.035	0.127	1.54
LP^1^	5.35–7.11	6.16**	0.38	0.21	6.2	0.027	-0.074	4.86–6.19	5.55	0.27	0.18	4.9	0.021	-0.104	1.11
LP^2^	5.38–6.83	6.23**	0.34	0.05	5.4	0.024	-0.123	5.02–6.47	5.60	0.30	0.13	5.4	0.023	-0.078	1.11
LP^3^	5.35–7.07	6.24**	0.38	0.26	6.1	0.026	-0.083	5.06–6.54	5.63	0.33	0.06	5.8	0.025	-0.055	1.11
LP^4^	5.25–7.14	6.38**	0.41	0.05	6.4	0.028	-0.062	5.08–6.84	5.85	0.40	0.12	6.8	0.029	0.001	1.09
LM^1^	5.70–7.56	6.70**	0.38	0.03	5.6	0.025	-0.119	5.36–7.38	6.29	0.46	0.16	7.3	0.032	0.038	1.07
LM^2^	2.89–6.45	5.31**	0.78	0.00	14.7	0.074	0.394	2.73–5.85	4.72	0.68	0.02	14.4	0.071	0.351	1.13
Widths of upper teeth															
WI^1^	3.64–5.19	4.37**	0.35	0.11	7.9	0.035	0.031	3.25–4.48	3.78	0.26	0.78	6.9	0.029	-0.014	1.16
WI^2^	3.94–5.48	4.69**	0.34	0.04	7.2	0.031	-0.002	3.50–4.79	4.10	0.27	0.37	6.5	0.028	-0.028	1.14
WI^3^	5.51–7.95	6.73**	0.55	0.27	8.2	0.036	0.053	4.28–6.08	5.20	0.46	0.99	8.9	0.040	0.107	1.30
WC^1^	7.57–11.38	9.18**	0.81	0.72	8.9	0.039	0.124	4.69–6.89	5.82	0.47	0.06	8.1	0.035	0.075	1.58
WP^1^	4.05–5.45	4.76**	0.36	0.19	7.6	0.033	0.018	3.71–5.06	4.33	0.29	0.90	6.7	0.029	-0.015	1.10
WP^2^	3.95–5.43	4.67**	0.35	0.07	7.6	0.033	0.015	3.68–4.86	4.19	0.26	0.67	6.3	0.027	-0.035	1.11
WP^3^	4.04–5.41	4.62**	0.31	2.49	6.7	0.029	-0.024	3.64–4.80	4.22	0.26	1.69	6.3	0.027	-0.035	1.09
WP^4^	3.93–5.09	4.56**	0.28	0.11	6.2	0.027	-0.049	3.68–4.81	4.20	0.27	3.05	6.4	0.028	-0.029	1.09
WM^1^	2.88–4.18	3.55*	0.24	0.27	6.8	0.030	-0.009	2.82–4.01	3.39	0.28	0.25	8.4	0.037	0.033	1.05
WM^2^	2.42–3.93	3.29**	0.32	0.29	9.7	0.044	0.088	2.28–3.54	2.97	0.28	1.10	9.3	0.041	0.051	1.11
Lengths of lower teeth															
LI_2_	2.63–3.51	3.17**	0.19	0.39	6.0*	0.026	-0.033	2.41–4.20	2.99	0.27	0.12	9.0	0.037	0.042	1.06
LI_3_	4.71–6.93	5.73**	0.50	1.68	8.7*	0.038	0.078	4.35–5.89	4.94	0.32	1.53	6.5	0.028	-0.021	1.16
LC_1_	10.10–15.54	12.23**	0.94	0.22	7.7	0.032	0.012	6.08–8.56	6.95	0.52	1.69	7.5	0.032	0.058	1.76
LP_1_	4.49–6.35	5.50**	0.35	0.93	6.5*	0.028	-0.048	4.68–5.57	5.08	0.24	0.91	4.8	0.021	-0.109	1.08
LP_2_	5.42–6.86	6.17**	0.30	2.08	4.9	0.021	-0.153	5.00–6.34	5.55	0.29	0.07	5.2	0.022	-0.090	1.11
LP_3_	5.87–7.46	6.75**	0.37	1.61	5.5	0.024	-0.127	5.39–6.69	6.02	0.31	0.07	5.1	0.022	-0.100	1.12
LP_4_	6.03–7.62	6.83**	0.38	0.27	5.6	0.024	-0.125	5.32–7.13	6.15	0.34	0.11	5.5	0.024	-0.078	1.11
LM_1_	6.20–8.33	7.24**	0.44	0.24	6.1	0.026	-0.098	5.89–7.68	6.65	0.36	0.04	5.4	0.023	-0.083	1.09
Widths of lower teeth															
WI_2_	2.78–3.84	3.19**	0.26	0.47	8.0	0.034	0.032	2.23–3.29	2.73	0.22	0.11	8.0	0.035	0.006	1.17
WI_3_	2.68–4.53	3.46**	0.37	0.53	10.7	0.046	0.125	2.25–3.17	2.77	0.23	2.17	8.3	0.037	0.016	1.25
WC_1_	5.68–8.99	7.44**	0.73	0.45	9.8	0.043	0.177	4.04–5.67	4.78	0.38	1.58	8.0	0.034	0.049	1.56
WP_1_	3.37–4.43	3.92**	0.26	1.30	6.7	0.029	-0.017	2.90–4.33	3.64	0.27	1.27	7.5	0.033	0.007	1.08
WP_2_	3.55–4.99	4.30**	0.29	0.83	6.7	0.029	-0.022	3.08–4.42	3.80	0.28	0.26	7.4	0.033	0.006	1.13
WP_3_	4.03–5.30	4.58**	0.28	1.73	6.1	0.026	-0.053	3.39–4.69	4.03	0.26	0.45	6.5	0.028	-0.027	1.14
WP_4_	4.20–5.43	4.70**	0.29	0.11	6.1	0.026	-0.053	3.39–4.67	4.17	0.26	1.25	6.3	0.028	-0.033	1.13
WM_1_	3.49–4.81	4.12**	0.29	0.24	7.0	0.030	-0.008	3.32–4.38	3.81	0.25	0.71	6.5	0.028	-0.026	1.08

Range, mean, SD, and RSD are given in millimeters, SDL is in log millimeters, ME and CV are in per cent. Asterisks indicate the means and CVs of males that differ significantly (*P ≤ 0.05, **P ≤ 0.001) from those of females according to Welch’s *t*-test (means) or *Z*-test (CVs) results. Statistical abbreviations and symbols are explained in Table 1. Abbreviations for tooth lengths and widths are expanded in Table 4.

**Table 7:** Statistics for Tooth Lengths and Widths in *Eumetopias jubatus*.

Variable	Males, *n* = 31							Females, *n* = 30							M/F
	Range	mean	SD	ME	CV	SDL	RSD	Range	mean	SD	ME	CV	SDL	RSD	
Lengths of upper teeth															
LI^1^	3.43–4.19	3.79**	0.21	0.97	5.5	0.024	0.019	3.05–3.62	3.33	0.15	0.26	4.4	0.019	-0.076	1.14
LI^2^	3.85–5.50	4.70**	0.38	0.09	8.2	0.036	0.127	3.59–4.71	4.14	0.29	0.19	7.0	0.030	0.028	1.13
LI^3^	9.91–13.14	11.70**	0.69	0.51	5.9	0.026	-0.093	7.35–9.92	8.40	0.69	1.32	8.2	0.035	0.224	1.39
LC^1^	18.83–27.26	21.84**	1.76	0.08	8.1	0.034	0.217	10.94–14.72	13.25	0.78	0.16	5.9	0.026	0.086	1.65
LP^1^	9.26–12.66	11.03**	0.67	0.08	6.1	0.027	-0.058	8.47–10.58	9.69	0.49	0.99	5.0	0.022	-0.036	1.14
LP^2^	11.98–15.16	13.34**	0.68	0.41	5.1	0.022	-0.227	10.66–12.62	11.53	0.46	1.53	4.0	0.017	-0.149	1.16
LP^3^	12.24–14.49	12.99**	0.60	0.37	4.6	0.020	-0.276	10.20–12.64	11.29	0.52	0.52	4.6	0.020	-0.080	1.15
LP^4^	11.63–14.20	12.68**	0.63	4.69	5.0	0.021	-0.229	10.15–12.44	11.25	0.53	0.38	4.7	0.020	-0.068	1.13
LM^1^	7.30–13.55	11.11*	1.12	0.11	10.0*	0.047	0.377	9.54–12.05	10.36	0.60	0.04	5.8	0.025	0.045	1.07
Widths of upper teeth															
WI^1^	5.40–6.77	6.06**	0.38	1.05	6.2	0.027	0.016	4.54–5.53	5.02	0.27	0.52	5.3	0.023	-0.034	1.21
WI^2^	6.34–8.54	7.47**	0.59	0.16	8.0*	0.035	0.128	5.55–6.74	6.04	0.29	0.70	4.8	0.021	-0.059	1.24
WI^3^	12.48–16.26	14.26**	0.92	0.08	6.4	0.028	-0.060	9.52–12.18	10.56	0.75	0.46	7.1	0.030	0.181	1.35
WC^1^	16.06–22.89	18.82**	1.36	0.02	7.2	0.031	0.045	10.09–12.01	10.98	0.56	0.15	5.1	0.022	-0.028	1.71
WP^1^	7.18–9.68	8.68**	0.60	0.38	6.9	0.030	0.039	6.58–8.42	7.67	0.39	0.25	5.1	0.022	-0.038	1.13
WP^2^	8.42–11.10	10.06**	0.64	0.20	6.3	0.028	-0.024	8.17–9.65	8.86	0.42	0.33	4.8	0.021	-0.061	1.14
WP^3^	9.07–11.49	10.16**	0.58	0.21	5.7	0.025	-0.086	7.74–9.77	8.90	0.45	0.01	5.1	0.023	-0.033	1.14
WP^4^	7.79–10.48	8.74**	0.62	0.30	7.1	0.030	0.064	6.64–8.78	7.79	0.47	0.59	6.0	0.026	0.033	1.12
WM^1^	6.04–7.67	6.80**	0.38	0.30	5.7	0.025	-0.030	5.32–6.94	6.10	0.42	0.79	6.9	0.030	0.066	1.12
Lengths of lower teeth															
LI_2_	4.22–6.04	5.17**	0.37	0.34	7.2	0.031	0.078	3.99–5.08	4.62	0.26	2.75	5.7	0.025	-0.018	1.12
LI_3_	8.25–10.76	9.08**	0.57	0.12	6.2	0.027	-0.021	7.17–8.74	8.07	0.47	0.81	5.9	0.026	0.025	1.13
LC_1_	20.06–28.98	23.64**	1.89	0.07	8.0*	0.034	0.210	11.71–14.69	12.98	0.67	0.33	5.2	0.022	-0.008	1.82
LP_1_	8.92–11.57	10.18**	0.61	0.19	6.0	0.026	-0.059	6.03–9.64	8.86	0.62	0.14	7.0	0.035	0.139	1.15
LP_2_	11.41–13.03	12.24**	0.49	0.22	4.0	0.017	-0.333	10.09–11.76	10.89	0.45	0.04	4.2	0.018	-0.128	1.12
LP_3_	12.98–15.53	14.15**	0.68	0.13	4.8	0.021	-0.292	11.14–13.30	12.36	0.54	0.25	4.3	0.019	-0.113	1.15
LP_4_	11.58–14.60	12.97**	0.84	0.03	6.4	0.028	-0.043	9.14–12.90	11.41	0.71	0.08	6.2	0.028	0.104	1.14
LM_1_	8.84–11.55	10.45**	0.71	0.05	6.8	0.030	0.024	7.81–10.96	9.49	0.71	0.15	7.5	0.033	0.199	1.10
Widths of lower teeth															
WI_2_	5.27–6.81	5.94**	0.40	0.37	6.7	0.029	0.050	4.20–5.46	4.96	0.30	0.42	6.1	0.027	0.003	1.20
WI_3_	5.82–8.76	6.67**	0.56	0.22	8.4	0.035	0.156	4.74–6.34	5.41	0.33	1.27	6.1	0.026	0.012	1.23
WC_1_	13.53–18.93	16.11**	1.29	0.18	8.0**	0.035	0.175	8.18–9.63	8.84	0.35	1.97	4.0	0.017	-0.134	1.82
WP_1_	6.75–8.70	7.68**	0.52	0.17	6.8	0.030	0.039	5.76–7.40	6.64	0.39	0.05	5.8	0.026	0.007	1.16
WP_2_	7.13–9.30	8.32**	0.49	0.85	5.9	0.026	-0.036	6.45–8.00	7.18	0.39	0.06	5.5	0.024	-0.013	1.16
WP_3_	8.41–10.60	9.43**	0.59	0.68	6.3*	0.027	-0.023	7.46–8.88	8.03	0.34	0.07	4.3	0.018	-0.101	1.18
WP_4_	7.44–10.10	8.77**	0.65	0.34	7.4	0.032	0.086	6.25–8.37	7.54	0.46	0.11	6.1	0.027	0.035	1.16
WM_1_	5.98–7.95	6.85**	0.46	0.16	6.8	0.029	0.045	5.40–6.66	6.02	0.34	0.71	5.7	0.025	-0.009	1.14

Range, mean, SD, and RSD are given in millimeters, SDL is in log millimeters, ME and CV are in per cent. Asterisks indicate the means and CVs of males that differ significantly (*P ≤ 0.05, **P ≤ 0.001) from those of females according to Welch’s *t*-test (means) or *Z*-test (CVs) results. Statistical abbreviations and symbols are explained in Table 1. Abbreviations for tooth lengths and widths are expanded in Table 4.

**Table 8:** Statistics for Tooth Lengths and Widths in *Histriophoca fasciata*.

Variable	Males, *n* = 62							Females, *n* = 86							M/F
	Range	mean	SD	ME	CV	SDL	RSD	Range	mean	SD	ME	CV	SDL	RSD	
Lengths of upper teeth															
LI^1^	1.27–2.28	1.80*	0.21	0.39	11.9	0.055	-0.033	1.12–2.27	1.71	0.19	0.09	10.9	0.049	-0.025	1.05
LI^2^	1.50–2.71	2.04	0.24	0.20	12.0	0.052	-0.022	1.42–2.80	1.98	0.23	0.26	11.5	0.050	-0.006	1.03
LI^3^	2.23–3.82	2.97	0.34	0.12	11.5*	0.050	-0.000	2.24–3.50	2.89	0.26	0.21	9.0	0.040	-0.045	1.03
LC^1^	4.54–6.62	5.52*	0.52	0.09	9.4	0.041	-0.030	4.18–6.39	5.29	0.40	0.12	7.6	0.033	-0.096	1.04
LP^1^	3.17–5.34	4.08	0.45	0.01	11.0	0.048	0.019	2.77–5.11	3.95	0.40	0.38	10.2	0.045	0.012	1.03
LP^2^	3.67–6.12	4.73	0.56	0.04	11.9	0.052	0.080	3.33–5.78	4.57	0.45	0.28	9.8	0.043	0.007	1.04
LP^3^	4.09–6.83	5.33	0.51	0.08	9.5	0.041	-0.026	3.76–6.33	5.30	0.52	0.09	9.9	0.044	0.025	1.01
LP^4^	4.19–6.94	5.24	0.52	0.02	10.0	0.043	-0.002	3.75–6.40	5.19	0.50	0.27	9.5	0.043	0.005	1.01
LM^1^	3.76–5.85	4.69*	0.46	0.07	9.8	0.043	-0.020	3.19–5.71	4.51	0.53	0.02	11.9	0.053	0.099	1.04
Widths of upper teeth															
WI^1^	1.60–2.88	2.12*	0.28	0.65	13.4	0.057	0.012	1.28–2.76	2.00	0.26	0.17	12.9	0.057	0.024	1.06
WI^2^	1.89–3.40	2.52	0.36	0.58	14.4	0.061	0.057	1.67–3.69	2.45	0.33	0.79	13.4	0.056	0.057	1.03
WI^3^	2.42–4.21	3.48	0.41	0.36	11.7	0.052	0.026	2.51–4.11	3.38	0.35	0.22	10.4	0.046	0.007	1.03
WC^1^	3.80–5.62	4.63*	0.40	0.55	8.6	0.037	-0.077	3.69–5.32	4.49	0.33	0.10	7.3	0.032	-0.104	1.03
WP^1^	2.51–4.21	3.24	0.39	0.29	12.1*	0.051	0.028	2.45–4.08	3.16	0.30	0.44	9.5	0.042	-0.026	1.03
WP^2^	2.44–4.47	3.19*	0.40	0.10	12.4*	0.054	0.037	2.50–3.84	3.07	0.28	0.58	9.2	0.040	-0.039	1.04
WP^3^	2.70–4.41	3.39	0.36	0.12	10.6	0.045	-0.017	2.43–3.94	3.30	0.28	1.25	8.5	0.038	-0.057	1.03
WP^4^	2.63–4.28	3.34	0.34	0.21	10.3	0.044	-0.028	2.44–4.09	3.30	0.30	1.34	9.1	0.041	-0.038	1.01
WM^1^	2.18–3.95	2.84	0.33	0.02	11.6	0.049	-0.002	1.88–3.66	2.74	0.33	0.13	12.0	0.053	0.034	1.04
Lengths of lower teeth															
LI_2_	0.62–1.53	1.13	0.19	0.43	16.9	0.080	-0.002	0.68–1.82	1.07	0.20	0.66	18.5	0.080	0.038	1.05
LI_3_	1.05–2.34	1.63	0.27	0.66	16.8	0.073	0.040	1.06–2.35	1.56	0.26	0.31	16.5	0.073	0.058	1.05
LC_1_	3.97–6.13	4.98*	0.48	0.11	9.5	0.042	-0.028	3.48–5.79	4.80	0.43	0.06	9.0	0.040	-0.025	1.04
LP_1_	2.78–4.42	3.57*	0.38	0.14	10.6	0.046	-0.012	1.66–4.25	3.42	0.40	0.35	11.8	0.057	0.056	1.05
LP_2_	4.05–6.29	5.01*	0.51	0.08	10.3	0.045	0.008	3.82–6.11	4.80	0.46	0.18	9.5	0.042	-0.003	1.04
LP_3_	4.73–7.76	5.65*	0.58	0.03	10.3	0.043	0.024	4.32–6.57	5.45	0.49	0.20	9.1	0.040	-0.016	1.04
LP_4_	4.62–7.28	5.67	0.59	0.02	10.4	0.045	0.028	3.81–6.68	5.58	0.58	0.05	10.4	0.047	0.058	1.02
LM_1_	3.62–7.64	5.45	0.63	0.02	11.5	0.051	0.084	3.28–6.49	5.33	0.69	0.06	12.9	0.060	0.187	1.02
Widths of lower teeth															
WI_2_	0.90–1.95	1.42	0.21	0.39	14.6	0.063	-0.009	0.89–2.06	1.44	0.21	0.72	14.8	0.065	0.024	0.98
WI_3_	1.22–2.94	1.75	0.28	0.58	15.9	0.067	0.037	1.21–2.37	1.77	0.23	0.57	12.7	0.056	0.009	0.99
WC_1_	3.26–5.13	4.01	0.37	0.58	9.3	0.040	-0.051	3.08–4.63	3.90	0.32	0.63	8.2	0.036	-0.067	1.03
WP_1_	2.22–3.75	2.86	0.33	0.15	11.7	0.050	0.002	1.48–3.47	2.76	0.30	0.28	10.9	0.051	0.006	1.03
WP_2_	2.79–4.27	3.49	0.35	0.06	10.1	0.044	-0.031	2.61–4.07	3.39	0.29	0.32	8.6	0.038	-0.054	1.03
WP_3_	2.87–4.57	3.61	0.37	0.17	10.1	0.044	-0.027	2.70–4.29	3.51	0.30	0.93	8.4	0.037	-0.060	1.03
WP_4_	2.88–4.54	3.52	0.35	0.10	9.8	0.042	-0.040	2.61–4.29	3.46	0.32	0.21	9.4	0.041	-0.027	1.02
WM_1_	2.53–4.16	3.14	0.33	0.14	10.5	0.045	-0.025	2.17–3.68	3.05	0.30	0.38	9.8	0.043	-0.021	1.03

Range, mean, SD, and RSD are given in millimeters, SDL is in log millimeters, ME and CV are in per cent. Asterisks indicate the means and CVs of males that differ significantly (*P ≤ 0.05; all *P* values exceed 0.001) from those of females according to Welch’s *t*-test (means) or *Z*-test (CVs) results. Statistical abbreviations and symbols are explained in Table 1. Abbreviations for tooth lengths and widths are expanded in Table 4.

**Table 9:** Statistics for Tooth Lengths and Widths in *Phoca largha*.

Variable	Males, *n* = 80							Females, *n* = 60							M/F
	Range	mean	SD	ME	CV	SDL	RSD	Range	mean	SD	ME	CV	SDL	RSD	
Lengths of upper teeth															
LI^1^	1.74–2.43	2.06**	0.13	0.16	6.3	0.028	-0.024	1.61–2.27	1.96	0.13	0.31	6.4	0.028	-0.047	1.05
LI^2^	2.05–2.85	2.52*	0.17	0.83	6.8	0.030	-0.015	2.03–2.88	2.42	0.19	0.36	8.0	0.035	-0.003	1.04
LI^3^	3.27–4.51	3.84**	0.27	1.33	7.0	0.031	-0.010	2.77–4.36	3.63	0.26	1.12	7.3	0.032	0.004	1.06
LC^1^	6.82–10.66	8.25**	0.71	0.12	8.6*	0.037	0.115	6.38–8.66	7.49	0.50	0.29	6.7	0.029	0.044	1.10
LP^1^	4.64–7.54	5.82*	0.52	0.35	9.0*	0.038	0.100	4.76–7.10	5.60	0.39	0.74	6.9	0.029	0.026	1.04
LP^2^	7.08–10.29	8.17**	0.61	0.05	7.5	0.032	0.024	6.56–8.57	7.46	0.48	0.08	6.4	0.028	0.018	1.09
LP^3^	7.50–10.30	8.60**	0.62	0.05	7.2*	0.031	-0.000	7.12–9.00	8.11	0.44	0.20	5.4	0.024	-0.053	1.06
LP^4^	6.32–9.59	8.20**	0.59	0.02	7.2	0.032	-0.002	6.62–8.80	7.82	0.48	0.09	6.1	0.027	0.004	1.05
LM^1^	6.58–9.34	7.93**	0.57	0.10	7.2	0.031	-0.006	6.60–8.73	7.60	0.52	0.06	6.8	0.030	0.052	1.04
Widths of upper teeth															
WI^1^	2.39–3.77	3.08**	0.30	0.44	9.6	0.042	0.069	2.25–3.36	2.74	0.21	1.38	7.8	0.034	0.001	1.12
WI^2^	2.78–4.59	3.61**	0.36	0.04	9.9	0.044	0.093	2.55–3.90	3.25	0.31	1.54	9.6	0.042	0.072	1.11
WI^3^	3.58–5.96	5.15**	0.38	1.92	7.5	0.034	0.010	3.94–5.47	4.67	0.36	0.45	7.8	0.034	0.049	1.10
WC^1^	5.07–8.10	6.95**	0.51	0.23	7.3	0.033	0.007	5.44–7.17	6.32	0.37	0.15	5.9	0.026	-0.025	1.10
WP^1^	3.32–4.77	4.00**	0.28	0.33	7.1	0.031	-0.008	3.27–4.34	3.75	0.26	0.32	7.1	0.030	-0.001	1.07
WP^2^	3.56–5.41	4.40**	0.33	0.16	7.6	0.033	0.014	3.50–4.54	4.06	0.25	0.58	6.1	0.027	-0.034	1.08
WP^3^	3.80–5.28	4.63**	0.28	0.28	6.1	0.027	-0.056	3.56–4.97	4.27	0.31	0.14	7.2	0.031	0.014	1.08
WP^4^	3.94–5.37	4.72**	0.28	0.37	6.0	0.026	-0.062	3.72–5.01	4.41	0.29	0.45	6.6	0.029	-0.009	1.07
WM^1^	3.77–5.22	4.51**	0.32	0.16	7.0	0.031	-0.013	3.60–5.01	4.24	0.31	0.10	7.3	0.032	0.017	1.06
Lengths of lower teeth															
LI_2_	1.68–2.29	1.92*	0.15	0.42	7.8	0.033	0.006	1.59–2.31	1.87	0.15	0.20	7.9	0.034	-0.020	1.03
LI_3_	2.07–2.93	2.57*	0.21	0.42	8.0	0.035	0.016	1.85–3.10	2.47	0.20	1.31	8.0	0.036	-0.001	1.04
LC_1_	5.84–9.19	7.53**	0.55	0.96	7.3	0.032	0.003	5.72–7.61	6.89	0.46	0.65	6.7	0.030	0.036	1.09
LP_1_	4.49–6.62	5.48**	0.39	0.98	7.0	0.030	-0.012	4.44–6.00	5.15	0.37	0.59	7.2	0.031	0.032	1.06
LP_2_	7.07–9.76	8.22**	0.58	0.10	7.1	0.030	-0.013	6.74–8.97	7.74	0.47	0.13	6.1	0.027	-0.001	1.06
LP_3_	7.79–10.42	9.08**	0.62	0.20	6.8	0.030	-0.035	7.40–9.85	8.49	0.49	0.01	5.7	0.025	-0.026	1.07
LP_4_	7.16–10.02	8.62**	0.59	0.02	6.8	0.030	-0.035	7.18–9.25	8.13	0.45	0.18	5.6	0.024	-0.042	1.06
LM_1_	8.08–10.96	9.35**	0.65	0.02	7.0	0.031	-0.020	7.68–9.82	8.90	0.50	0.45	5.6	0.024	-0.035	1.05
Widths of lower teeth															
WI_2_	1.79–2.68	2.17**	0.17	0.37	8.0	0.035	0.012	1.75–2.61	2.01	0.15	1.38	7.7	0.033	-0.021	1.08
WI_3_	2.22–3.09	2.60**	0.18	0.64	7.1	0.031	-0.008	1.99–2.86	2.41	0.17	0.65	7.0	0.031	-0.026	1.08
WC_1_	5.18–7.60	6.21**	0.44	0.52	7.2	0.031	-0.005	4.98–6.35	5.63	0.33	2.23	5.8	0.025	-0.036	1.10
WP_1_	3.46–4.67	3.96**	0.27	0.64	6.7	0.029	-0.024	2.88–4.24	3.69	0.28	1.22	7.5	0.033	0.013	1.07
WP_2_	4.09–5.55	4.78**	0.33	0.42	6.9	0.030	-0.020	3.80–5.14	4.47	0.30	0.83	6.7	0.029	-0.002	1.07
WP_3_	4.10–5.61	4.90**	0.31	0.19	6.4	0.028	-0.044	3.79–5.20	4.55	0.31	2.53	6.8	0.030	0.001	1.08
WP_4_	4.03–5.46	4.67**	0.30	0.11	6.5	0.029	-0.035	3.69–4.98	4.35	0.28	1.38	6.4	0.028	-0.017	1.07
WM_1_	3.75–5.29	4.68**	0.32	0.51	6.8	0.030	-0.022	3.60–5.03	4.34	0.31	0.24	7.2	0.032	0.017	1.08

Range, mean, SD, and RSD are given in millimeters, SDL is in log millimeters, ME and CV are in per cent. Asterisks indicate the means and CVs of males that differ significantly (*P ≤ 0.05, **P ≤ 0.001) from those of females according to Welch’s *t*-test (means) or *Z*-test (CVs) results. Statistical abbreviations and symbols are explained in Table 1. Abbreviations for tooth lengths and widths are expanded in Table 4.

In all species, I^1^ was smaller than I^2^, I^2^ was smaller than I^3^, and I_2_ was smaller than I_3_. The incisors had the smallest mean mesiodistal length in all species except I^3^ in male *E*. *jubatus* and I_3_ in male *C*. *ursinus*. I^1^ in all species except *C*. *ursinus*, I^2^ in all species except *C*. *ursinus* and male *E*. *jubatus*, and both lower incisors in all species had the smallest mean vestibulolingual width (Figs 2A, 3A, 4A and 5A).

The canines were the largest teeth in both the upper and lower dentitions in all species (Fig 1). The canines had the greatest mean vestibulolingual width in all species and the greatest mean mesiodistal length in *C*. *ursinus* and *E*. *jubatus*; C^1^ also had the greatest mean length in male *H*. *fasciata*. C^1^ in female and C_1_ in female and male *H*. *fasciata* as well as both canines in both sexes of *P*. *largha* had a smaller mean length than one or more postcanines in the toothrow (Figs 2A, 3A, 4A and 5A). The postcanines that significantly (*P* ≤ 0.05, Welch’s *t*-test) exceeded the canine of the toothrow in mean length were P_3_, P_4_, and M_1_ in male and female *H*. *fasciata*; P^3^, P_2_, P_3_, P_4_, and M_1_ in male and female *P*. *largha*; and P^4^ in female *P*. *largha*.

The postcanines were largely of similar size within the toothrow (Fig 1) and partly varied in sequence according to size within species. In *C*. *ursinus*, M^1^ was the mesiodistally longest upper postcanine in most specimens, M^2^ was the shortest in most specimens, and the upper premolars were vestibulolingually broader than the upper molars (with M^1^ mostly broader than M^2^). M_1_ was in most cases the longest lower postcanine, P_1_ was the shortest and in most cases the narrowest, P_2_ was the second shortest, and P_4_ was in most cases the broadest (Fig 2A).

In *E*. *jubatus*, P^2^ was the longest upper postcanine in most specimens, whereas P^1^ or, more rarely, M^1^ was the shortest. P^2^ and P^3^ were broader than other upper postcanines. M^1^ was the narrowest upper postcanine. P_3_ was the longest and broadest lower postcanine. P_1_ and M_1_ were shorter than other lower postcanines (P_1_ was in most cases shorter than M_1_). M_1_ was in most cases the narrowest lower postcanine, and P_1_ was in most cases the second narrowest (Fig 3A).

In *H*. *fasciata*, P^3^ and P^4^ were longer and broader than other upper postcanines in most specimens. P^1^ was in most cases the shortest upper postcanine, and M^1^ was in most cases the narrowest. P_3_ and P_4_ were longer and broader than other lower postcanines in most specimens. P_1_ was the shortest and in most cases the narrowest lower postcanine, P_2_ was in most cases the second shortest, and M_1_ was in most cases the second narrowest (Fig 4A).

In *P*. *largha*, P^3^ was the longest upper postcanine in most specimens, P^4^ was the broadest in most specimens, and P^1^ was the shortest and in most cases the narrowest. P_3_ and M_1_ were longer than other lower postcanines in most specimens (M_1_ was in most cases longer than P_3_). P_3_ was also in most cases the broadest lower postcanine, and P_1_ was both the shortest and the narrowest (Fig 5A).

### Measurement error

MEs ranged 0.00–3.75% in *C*. *ursinus* (Table 6), 0.01–4.69% in *E*. *jubatus* (Table 7), 0.01–1.34% in *H*. *fasciata* (Table 8), and 0.01–2.53% in *P*. *largha* (Table 9). ME and mean variable size were negatively correlated in all species, but this correlation was significant only in *H*. *fasciata* and female *P*. *largha* (Table 10).

**Table 10:** Pearson’s Product Moment Correlation Coefficients and Their Statistical Significance among the Arithmetic Mean, Measurement Error, and Variation and Sexual Dimorphism Indices for Tooth Lengths and Widths in Pinniped Species.

Comparison	*Callorhinus ursinus*		*Eumetopias jubatus*		*Histriophoca fasciata*		*Phoca largha*	
	Males	Females	Males	Females	Males	Females	Males	Females
mean–ME	-0.10	-0.30	-0.07	-0.23	-0.62**	-0.37*	-0.31	-0.38*
mean–CV	0.03	-0.21	0.06	-0.20	-0.76**	-0.61**	-0.10	-0.71**
mean–SDL	0.00	-0.20	0.03	-0.19	-0.76**	-0.57**	-0.13	-0.70**
mean–M/F	0.75**	0.42*	0.75**	0.37*	-0.01	-0.04	0.05	0.00
CV–SDL	0.99**	0.99**	0.99**	0.99**	0.99**	0.99**	0.99**	1.00**
CV–RSD	0.97**	0.96**	0.96**	0.95**	0.52*	0.67**	0.93**	0.60**
CV–M/F	0.36*	0.24	0.34*	-0.09	-0.03	0.05	0.33	0.11
SDL–M/F	0.29	0.20	0.29	-0.12	0.03	0.05	0.35*	0.12
RSD–M/F	0.39*	0.38*	0.36*	0.01	-0.00	-0.03	0.31	0.24

Statistically significant coefficients according to Student’s *t*-test results are indicated by asterisks (*P ≤ 0.05, **P ≤ 0.001). Abbreviations are explained in Table 1.

### Variation indices

**Fig 8:**
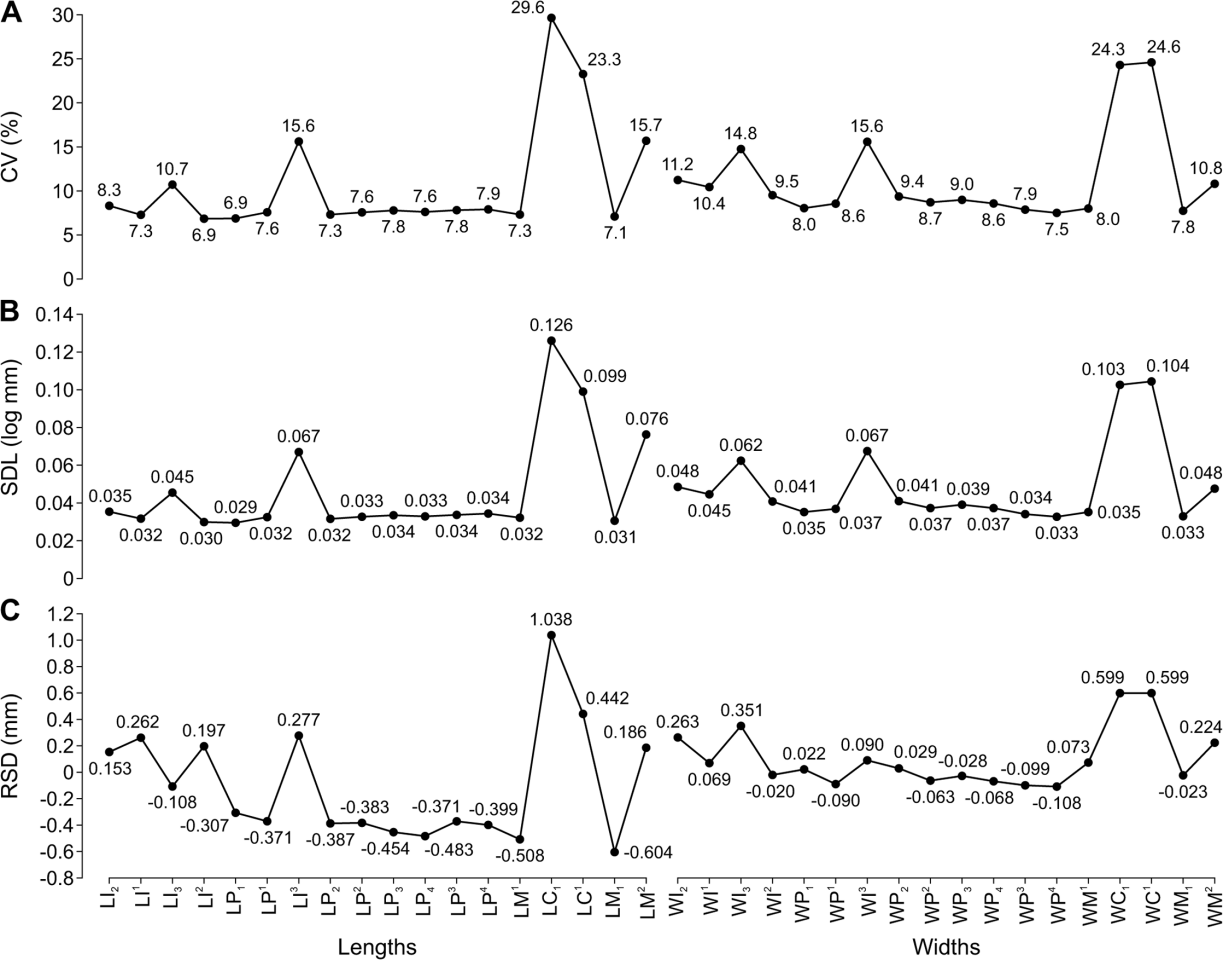
Variation Indices for Tooth Lengths and Widths Varied according to the Tooth Eruption Sequence in Pooled Sexes of *Callorhinus ursinus*. A, coefficient of variation (CV); B, standard deviation of log-transformed data (SDL); C, residual of standard deviation on arithmetic mean (RSD). Variables are ordered according to the sequence of tooth eruption [47] from the first (left) to the last (right) erupting tooth. Numbers at dots are the values of indices for the corresponding variables. Abbreviations for tooth lengths and widths are explained in Table 4.

CVs and SDLs were in most cases highest in *H*. *fasciata*, in most cases lowest in *E*. *jubatus*, and in most cases intermediate in *C*. *ursinus* and *P*. *largha* (Figs 6B, 6C, 7B and 7C). Both the highest and lowest RSDs were most frequent in *E*. *jubatus* (Figs 6D and 7D). *H*. *fasciata* had the widest range of CVs, *C*. *ursinus* had the widest range of SDLs, and *E*. *jubatus* had the widest range of RSDs; *P*. *largha* showed the narrowest range for all of these indices. Specifically, CVs, SDLs, and RSDs, respectively, ranged 4.8–14.7%, 0.021–0.074 log mm, and -0.153–0.394 mm in *C*. *ursinus* (Fig 2B–2D, Table 6); 4.0–10.0%, 0.017–0.047 log mm, and -0.333–0.377 mm in *E*. *jubatus* (Fig 3B–3D, Table 7); 7.3–18.5%, 0.032–0.080 log mm, and -0.104–0.187 mm in *H*. *fasciata* (Fig 4B–4D, Table 8); and 5.4–9.9%, 0.024–0.044 log mm, and -0.062–0.115 mm in *P*. *largha* (Fig 5B–5D, Table 9). As expected, CVs and SDLs were in most cases higher when males and females were pooled (for *C*. *ursinus*, compare Table 6 and Fig 8).

**Fig 9:**
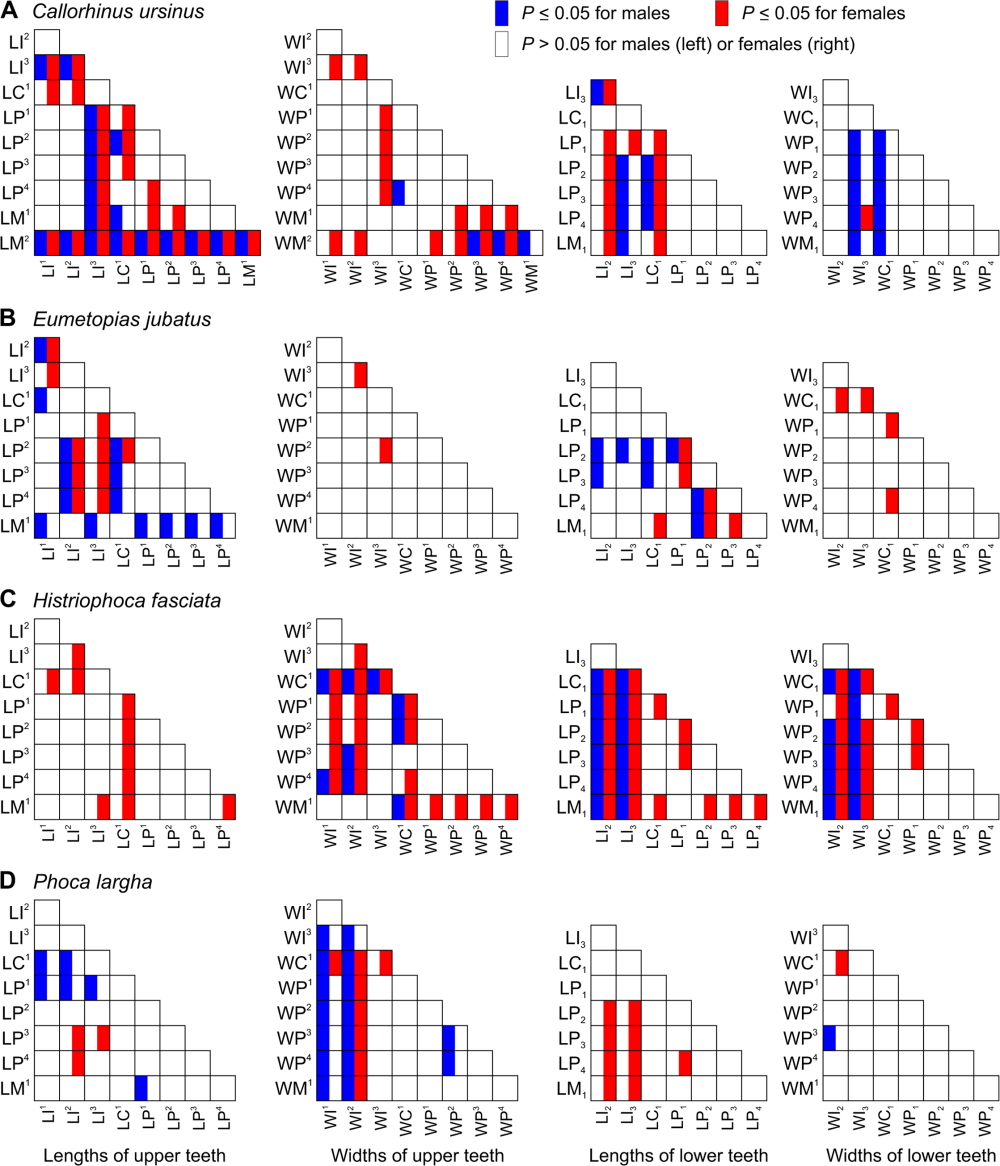
Statistical Significance of Pairwise Differences between the Coefficients of Variation for Tooth Lengths or Widths within the Toothrow in Pinniped Species. *P* values come from the *Z*-test. The coefficients of variation are shown in Tables 6–9. Abbreviations for tooth lengths and widths are explained in Table 4.

When the variation indices were compared among the classes of teeth within the toothrow, the indices for incisors were largely relatively high. The incisor indices varied from the lowest (CV, SDL, and RSD in *P*. *largha*, and RSD in *H*. *fasciata*) to the highest (CV, SDL, and RSD in all species) in the toothrow (Figs 2B–2D, 3B–3D, 4B–4D and 5B–5D). Of a total of 10 incisor size variables, six variables (WI^1^, WI^2^, LI_2_, WI_2_, LI_3_, and WI_3_) in one or both sexes of *H*. *fasciata*, five variables (LI^3^, WI^3^, LI_2_, LI_3_, and WI_3_) in one or both sexes of *C*. *ursinus*, four variables (WI^1^, WI^2^, LI_2_, and LI_3_) in one or both sexes of *P*. *largha*, and one variable (LI^3^) in female *E*. *jubatus* had their CVs significantly higher than most CVs for the corresponding variables (lengths or widths, respectively) within the toothrow (Fig 9).

In comparison to the variation indices for other tooth classes, those for canines were high in *C*. *ursinus*, male *E*. *jubatus*, and mostly also in male *P*. *largha*; low in *H*. *fasciata*; and from low to high in female *E*. *jubatus* and female *P*. *largha*. The canine indices varied from the lowest (CV, SDL, and RSD in female *E*. *jubatus*, female and male *H*. *fasciata*, and female *P*. *largha*) to the highest (CV, SDL, and RSD in male *E*. *jubatus* and RSD in both sexes of *C*. *ursinus* and *P*. *largha*) in the toothrow (Figs 2B–2D, 3B–3D, 4B–4D and 5B–5D). CVs for LC^1^, LC_1_, and WC_1_ in female or male *C*. *ursinus* were significantly higher while those for WC_1_ in female *E*. *jubatus* and LC^1^, WC^1^, LC_1_, and WC_1_ in female or female and male *H*. *fasciata* were significantly lower than most CVs for other tooth lengths or widths, respectively, in the toothrow (Fig 9).

The variation indices for postcanines mostly tended to progressively increase or decrease from the first premolar to the last molar or decrease from both the first premolar and last molar to one of the intermediary postcanines. These indices varied from the lowest (CV, SDL, and RSD in *C*. *ursinus*, *E*. *jubatus*, and *P*. *largha*) to the highest (CV, SDL, and RSD in all species) in the toothrow (Figs 2B–2D, 3B–3D, 4B–4D and 5B–5D). CV for LM^2^ significantly exceeded all other CVs for tooth lengths within the toothrow in both sexes of *C*. *ursinus*. CVs for two other variables (LP^1^ and WM^2^) in female *C*. *ursinus*, two variables (LM^1^ and LP_2_) in male *E*. *jubatus*, and six variables (WM^1^, LP_1_, WP_1_, LP_2_, LP_3_, and LM_1_) in female *H*. *fasciata* differed significantly from most CVs for other tooth lengths or widths, respectively, in the toothrow (Fig 9).

CV and SDL were significantly correlated with mean variable size only in *H*. *fasciata* and female *P*. *largha*. There was a significant positive correlation between CV and both SDL and RSD (Table 10). The weakest relationship between CV and RSD was observed in male *H*. *fasciata* (*r* = 0.52), female *H*. *fasciata* (*r* = 0.67), and female *P*. *largha* (*r* = 0.60). However, when the incisor variables were excluded from the comparison, *r* for this relationship increased to 0.89 in male *H*. *fasciata*, 0.98 in female *H*. *fasciata*, and 0.68 in female *P*. *largha*, all of these values being highly significant at *P* < 0.0003 according to Student’s *t*-test results.

**Fig 10:**
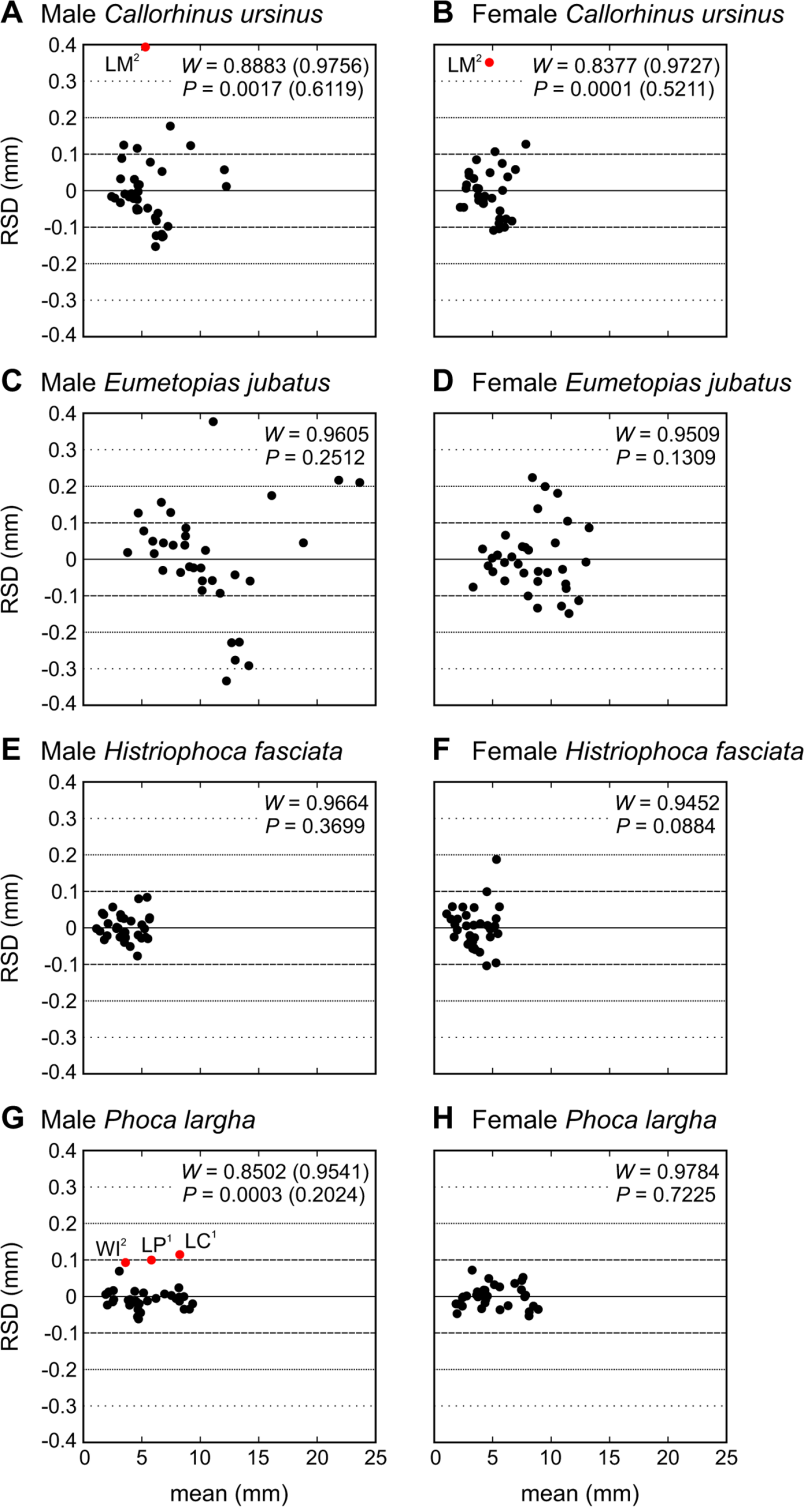
Relationship between the Residual of Standard Deviation on Arithmetic Mean (RSD) and the Arithmetic Mean (mean) for Tooth Lengths and Widths in Pinniped Species. Data from all teeth (Tables 6–9). Inset numbers represent the results of the Shapiro–Wilk test (*W*) and, under this test, the probability of wrongly rejecting the null hypothesis that the data are normally distributed (*P*). Numbers in parentheses refer to all data except outliers from an otherwise normal distribution. These outliers are highlighted in red and labeled with the variable abbreviation (explained in Table 4).

Plotting RSD against mean variable size revealed outliers from an otherwise normal distribution in both sexes of *C*. *ursinus* (LM^2^) and male *P*. *largha* (WI^2^, LC^1^, and LP^1^). No such outliers occurred in female *P*. *largha* and either sex of *E*. *jubatus* and *H*. *fasciata* (Fig 10).

### Sexual dimorphism

All mean variables were significantly higher in males than in females in *C*. *ursinus*, *E*. *jubatus*, and *P*. *largha* (Tables 6, 7 and 9). In *H*. *fasciata*, 10 mean variables were significantly higher and 22 mean variables were insignificantly higher in males, whereas two mean variables (WI_2_ and WI_3_) were insignificantly higher in females (Table 8). Most variables had their CVs higher in males than in females in all species (Figs 2B, 3B, 4B and 5B), although the CVs of males and females differed significantly for only five variables in *E*. *jubatus* (Table 7) and three variables in *C*. *ursinus* (Table 6), *H*. *fasciata* (Table 8), and *P*. *largha* (Table 9). SDLs were also mostly higher in males than in females in all species (Figs 2C, 3C, 4C and 5C). RSDs were mostly higher in males than in females in *E*. *jubatus* (Fig 3D), as frequently higher as lower in males than in females in *C*. *ursinus* (Fig 2D), and mostly lower in males than in females in *H*. *fasciata* and *P*. *largha* (Figs 4D and 5D).

M/Fs ranged 1.05–1.76 in *C*. *ursinus* (Table 6), 1.07–1.82 in *E*. *jubatus* (Table 7), 0.98–1.06 in *H*. *fasciata* (Table 8), and 1.03–1.12 in *P*. *largha* (Table 9). Most variables had the highest M/F in *E*. *jubatus*, the second highest in *C*. *ursinus*, the third highest in *P*. *largha*, and the lowest in *H*. *fasciata*. When M/Fs were compared within species, the M/Fs of canines were highest and those of I^3^ second highest in *C*. *ursinus* and *E*. *jubatus* but not in *H*. *fasciata* and *P*. *largha*, where the M/Fs of canines were within the 19 and eight highest M/Fs, respectively, and the M/Fs of I^3^ were within the 25 and 26 highest, respectively (Tables 6–9).

There was a significant positive correlation between M/F and mean variable size in *C*. *ursinus* and *E*. *jubatus* but not in *H*. *fasciata* and *P*. *largha* (Table 10). There was a moderate but significant positive correlation between M/F and CV in male *C*. *ursinus* and male *E*. *jubatus*, between M/F and SDL in male *P*. *largha*, and between M/F and RSD in both sexes of *C*. *ursinus* and male *E*. *jubatus*. The weakest relationship between M/F and the variation indices was in *H*. *fasciata* (Table 10).

### Effect of tooth eruption sequence

There was a significant positive correlation between the sequence of tooth eruption and both mean and SD for the lengths of teeth in *C*. *ursinus* but not for the widths. There was no significant correlation between the tooth eruption sequence and CV, SDL, RSD, or M/F (Table 11). The variation indices were not progressively higher when ordered according to the sequence of tooth eruption (Fig 8).

**Table 11:** Spearman’s Rank Correlation Coefficients and Their Statistical Significance between the Sequence of Tooth Eruption and the Arithmetic Mean, Standard Deviation, and Variation and Sexual Dimorphism Indices for Tooth Lengths and Widths in *Callorhinus ursinus*.

Comparison	Tooth lengths						Tooth widths					
	Males		Females		Males and females		Males		Females		Males and females	
	*r*_*s*_	*P*	*r*_*s*_	*P*	*r*_*s*_	*P*	*r*_*s*_	*P*	*r*_*s*_	*P*	*r*_*s*_	*P*
Eruption sequence–mean	0.74*	0.000	0.76*	0.000	0.75*	0.000	0.17	0.487	0.32	0.197	0.25	0.310
Eruption sequence–SD	0.66*	0.004	0.88*	0.000	0.67*	0.003	0.02	0.928	0.40	0.104	-0.18	0.482
Eruption sequence–CV	0.12	0.638	0.21	0.398	0.32	0.197	-0.05	0.831	0.04	0.876	-0.18	0.462
Eruption sequence–SDL	0.12	0.621	0.23	0.366	0.32	0.191	-0.07	0.799	0.04	0.869	-0.16	0.530
Eruption sequence–RSD	-0.06	0.805	0.29	0.242	-0.23	0.357	-0.06	0.824	0.11	0.668	-0.01	0.967
Eruption sequence–M/F	0.39	0.110	0.39	0.110	0.39	0.110	-0.26	0.294	-0.26	0.294	-0.26	0.294

The sequence of tooth eruption follows Scheffer and Kraus [47] and is: I_2_, I^1^, I_3_, I^2^, P_1_, P^1^, I^3^, P_2_, P^2^, P_3_, P_4_, P^3^, P^4^, M^1^, C_1_, C^1^, M_1_, and M^2^ (tooth symbols expanded in Table 5). Statistically significant coefficients at *P* ≤ 0.05 are indicated by an asterisk. *P* values derive from algorithm AS 89 [48]. Statistical abbreviations and symbols are explained in Table 1.

## Discussion and Conclusions

### Reliability and usefulness of variation indices

Although variable size ranged widely in our study, most MEs were low (<0.5%) and there was mostly no significant negative correlation between CV and mean variable size. CV, SDL, and the size-independent RSD varied similarly along the toothrow and were significantly correlated in all species. These results suggest that in most cases CV measured variation reliably.

In all species, CV and SDL showed similar levels of correlation with mean variable size, varied almost identically along the toothrow, and were very strongly correlated with each other (*r* = 0.99–1.00). This agrees with previous observations from land carnivorans [13–15] and suggests the redundancy of SDL with CV.

Even though independent of size and apparently mostly reliable, RSD was not fully reliable in both sexes of *C*. *ursinus* and male *P*. *largha*, as indicated by outliers from an otherwise normal distribution in the plots of this index against mean variable size. This problem of RSD was previously noticed by Polly [12], who argued that such outliers caused RSDs of large teeth to appear artificially low. Because such outliers represent relatively highly variable variables, we conclude that RSD can reliably measure variation when all variables are similarly variable, but this index may be misleading when one or more variables are markedly more variable than others. Another drawback that reduces the utility of RSD for measuring variation is that in contrast to CV and SDL, RSD is not comparable between different groups of organisms (e.g., species or sexes) if obtained from different regression analyses and that the way how RSD is calculated covers potential differences in variation between the compared groups of organisms (RSDs from each regression analysis are both positive and negative and center around zero).

### Tooth size variation in pinnipeds

#### Differential variation along the toothrow and among species

Contrary to the hypothesis of Polly [12] that size variation among mammalian teeth is relatively homogeneous both within and among species except highly variable canines in some species, our results show that size variation among pinniped teeth can considerably differ both within and among species. Even though this variation was relatively homogeneous in *P*. *largha*, it was clearly heterogeneous in *C*. *ursinus*, *E*. *jubatus*, and *H*. *fasciata*. Not only canines but also incisors were relatively highly variable in most cases although the canines of *H*. *fasciata* were less variable than other teeth of the toothrow. Incisor and canine variables were partly difficult to measure, which could result in artificial inflation of their variation indices, but any relevant contribution from this potential source was not supported by the MEs of these variables, which were mostly lower than 1%. Moreover, differences in size variation were observed among postcanines. Probably most remarkable is the relatively high variation in the size of the most distal upper postcanines in both otariids, *C*. *ursinus* (M^2^) and *E*. *jubatus* (M^1^).

Our results from CV and SDL indicate that the dentition of *H*. *fasciata* is the most variable in size, that of *E*. *jubatus* is the least variable, and those of *C*. *ursinus* and *P*. *largha* are at intermediate levels of the overall size variation. Admittedly, there was a significant negative correlation between CV and mean variable size in both sexes of *H*. *fasciata* and female *P*. *largha*, which suggests that the values of this index might be artificially inflated in these species due to the size-related bias revealed by Polly [12]. However, contrary to this suggestion, CV was mostly lower in females than in males in both species although all (*P*. *largha*) or almost all (*H*. *fasciata*) mean variables were higher in males than in females. Furthermore, there was no significant negative correlation between CV and mean variable size in male *P*. *largha*, *C*. *ursinus*, or *E*. *jubatus*.

A comparison of CVs for the lengths of lower postcanines between *Pagophilus groenlandicus* (6.6–8.8% [40]) and *H*. *fasciata* (9.1–12.9%) suggests that the dentition of the former species is less variable in size. The CVs of 9.9% (males) and 9.5% (females) reported for the length of P_3_ in *Pusa hispida* [40] are similar to those in *H*. *fasciata* (10.3 and 9.1%, respectively). In turn, CVs for the length and width of C^1^ recorded from male Antarctic (*Arctophoca gazella*; 5.7% and 8.0%, respectively) and subantarctic (*Arctophoca tropicalis*; 8.1% and 8.8%, respectively) fur seals [49] are similar to or lower than those in the males of species examined here (8.0–9.4% and 7.2–8.9%, respectively).

#### Sexual dimorphism

Our results indicate that sexual size dimorphism is the most pronounced for the dentition of *E*. *jubatus*, the second most pronounced for the dentition of *C*. *ursinus*, the third most pronounced for the dentition of *Phoca largha*, and the least pronounced for the dentition of *H*. *fasciata*, with teeth being, on average, larger in males than in females in all species. Miller et al. [40] reported that the lower postcanines of *Pagophilus groenlandicus* and P_3_ of *Pusa hispida* were, on average, also larger in males. It has also been noted that the canines of the northern and southern elephant seals (*Mirounga angustirostris* and *M*. *leonina*) were larger in males [50–52].

The canines and, to a lesser degree, I^3^ were the most sexually dimorphic teeth in *C*. *ursinus* and *E*. *jubatus*, where the observed ranges of canine variables did not overlap between sexes. The canines of these species were larger and more sexually dimorphic relative to other teeth than those of *H*. *fasciata* and *Phoca largha*. Moreover, there was a positive relation between sexual dimorphism and tooth size in both otariids, but no such a relationship was observed in the two phocids.

### Comparison with nonpinniped carnivorans

**Fig 11:**
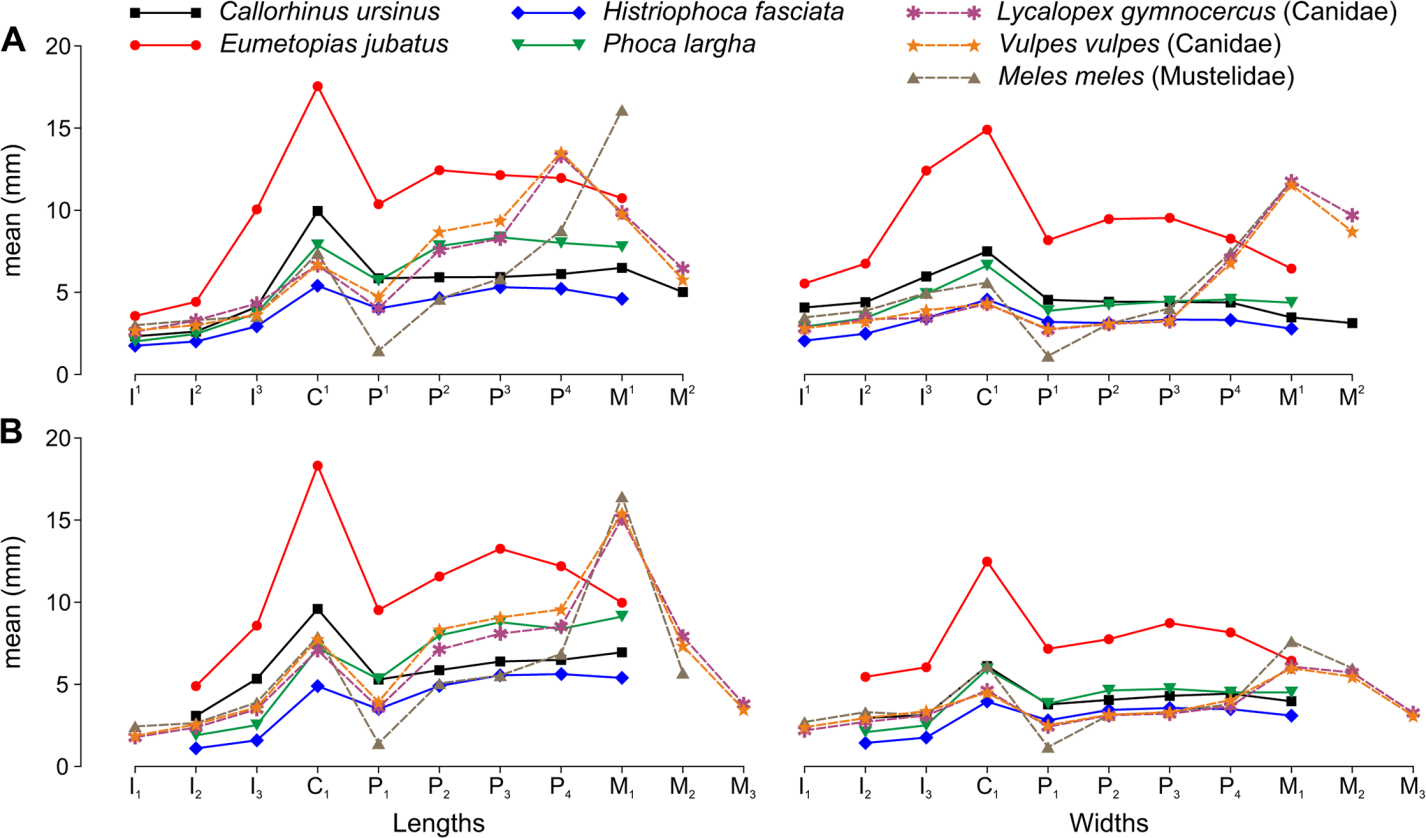
Arithmetic Mean for Tooth Lengths and Widths Varied along the Toothrow and among Pinniped and Other Carnivoran Species. A, upper teeth; B, lower teeth. Lengths are mesiodistal, widths are vestibulolingual. Tooth symbols are explained in Table 5. Family affiliations are indicated for nonpinniped species. Arithmetic mean (mean) values are averaged between sexes based on data from this study (pinnipeds) and previous investigations (*Lycalopex gymnocercus*, pampas fox [F. J. Prevosti, pers. comm., 26 February 2013]; *Vulpes vulpes*, red fox [13]; *Meles meles*, European badger [25]) except for P^1^ and P_1_ of *M*. *meles*, where the values are from females [53].

A comparison of how the size of teeth changes with position along the toothrow between pinnipeds and other carnivorans (Fig 11) shows that the incisors and canines exhibit a similar variation, whereas the postcanines exhibit a different variation. Unlike the postcanines of land carnivorans, the pinniped postcanines are mostly very similar in size and, as indicated by our measurements, some can vary in sequence according to size within species.

**Fig 12:**
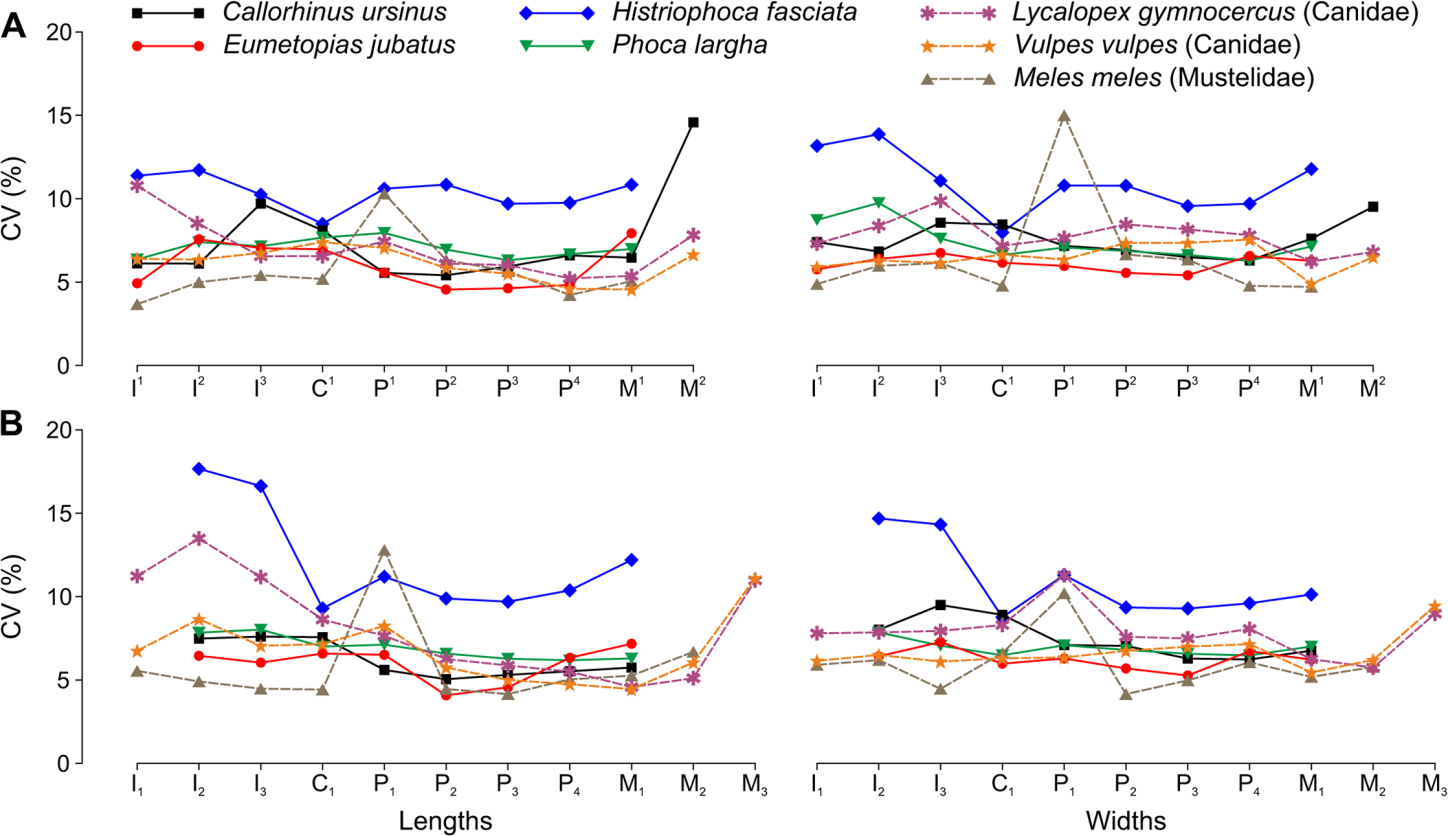
Coefficient of Variation for Tooth Lengths and Widths Varied along the Toothrow and among Pinniped and Other Carnivoran Species. A, upper teeth; B, lower teeth. Lengths are mesiodistal, widths are vestibulolingual. Tooth symbols are explained in Table 5. Family affiliations are indicated for nonpinniped species. Coefficient of variation (CV) values are averaged between sexes based on data from this study (pinnipeds) and previous investigations (*Lycalopex gymnocercus* [F. J. Prevosti, pers. comm., 25 October 2012], *Vulpes vulpes* [13], *Meles meles* [25]) except for P^1^ and P_1_ of *M*. *meles*, where CVs are from females [53]. For CVs from other land carnivoran species or other populations of the same land carnivoran species, see [12, 15, 16, 21, 24, 32, 36, 54–60]. Note that CVs based on pooled sexes [12, 14, 16, 21, 36, 54, 55, 59] can be higher than for either sex separately (they are totally or mostly higher when derived from sexually dimorphic species; compare CVs in Table 6 and Fig 8) and therefore comparing them directly with our averaged CVs can be misleading.

Contrary to the hypothesis of Miller et al. [40] that the postcanines of pinnipeds are more variable in size than those of land carnivorans due to evolutionary simplification of morphology, our results indicate that the pinniped dentitions represent a wide spectrum of the levels of size variation ranging from a relatively low variation as in land carnivorans to a high variation (Fig 12). Furthermore, although the postcanines of both otariids were mostly simpler in form than those of the phocids (Fig 1), the latter were mostly more variable in size (Fig 12).

### Testing hypotheses that explain differential size variation along the toothrow

#### The hypothesis invoking relative tooth position in a developmental field

Our results have not shown a consistent pattern to support this hypothesis. Whereas some of the results fit the predictions of the hypothesis (e.g., all variation indices for the lengths of upper teeth in male *E*. *jubatus* progressively increased in value from the central part to the mesial and distal ends of the postcanine toothrow; Fig 3B–3D), other results do not (e.g., all variation indices for the lengths of upper teeth in female *C*. *ursinus* progressively increased in value from the mesial to the distal end of the postcanine toothrow; Fig 2B–2D).

#### The hypothesis invoking relative tooth occlusal complexity

According to this hypothesis, the level of size variation in teeth is inversely proportional to their occlusal complexity [20]. This means that teeth that have similar occlusal complexity should be similarly variable in size. Our findings mostly negate this prediction. Even though teeth with similar occlusal complexity in most cases showed a similar size variation in *Phoca largha*, such teeth in most cases differed in size variation in *C*. *ursinus*, *E*. *jubatus*, and *H*. *fasciata*.

#### The hypothesis invoking the relative timing of tooth formation and sexually dimorphic hormonal activity

Our results do not support this hypothesis. In *C*. *ursinus* we have found neither a significant positive correlation between the sequence of tooth eruption and CV, SDL, or RSD nor a significant relationship between this sequence and M/F, and the teeth ordered according to the eruption sequence have not shown progressively higher variation indices for pooled males and females.
